# Dipole-Moment Modulation in New Incommensurate Ferrocene

**DOI:** 10.1021/acs.jpclett.3c00215

**Published:** 2023-03-23

**Authors:** Andrzej Katrusiak, Michalina Rusek, Michal Dušek, Václav Petříček, Marek Szafrański

**Affiliations:** † Faculty of Chemistry, Adam Mickiewicz University, Uniwersytetu Poznańskiego 8, 61-614 Poznan, Poland; ‡ Institute of Physics, Czech Academy of Sciences, Na Slovance 2, 182 21 Praha, Czech Republic; § Faculty of Physics, Adam Mickiewicz University, Uniwersytetu Poznańskiego 2, 61-614 Poznan, Poland

## Abstract

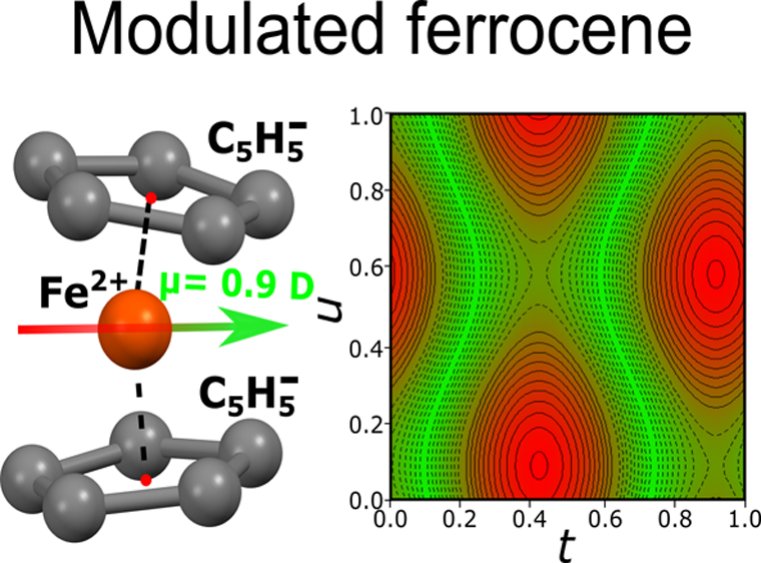

Despite 70 years of research on metallocenes and their applications, there are still unresolved regions in its phase diagram of the prototypic sandwich compound, ferrocene Fe^2+^[C_5_H_5_]^−^_2_ (FeCp_2_), and its molecular 5-fold symmetry cannot be reconciled with the dielectric response of this crystal. We found a new phase I″ of ferrocene, which reveals the relationships between the molecular conformation, intermolecular interactions, and electric permittivity of this compound. Between 172.8 and 163.5 K, the conformational disorder of ferrocene molecules transforms into the incommensurate modulation. The structure of phase I″ is described in the (3+2)-dimensional superspace, where the molecular conformations, rotations and inclinations of the Cp rings, molecular tilts, and displacements of the Fe^2+^ cations, as well as the CH···π bonds in the crystal environment, are modulated. These geometric changes combine into the FeCp_2_ bending distortion, breaking the 5-fold symmetry and generating waves of molecular dipole moments with their amplitudes approaching 4 × 10^–30^ C·m.

The discovery of ferrocene^1,2^ sparked the organometallic revolution^3,4^ and today this seminal compound is not only one of the commonly known chemical reagents and precursors, but it and its derivatives find versatile practical applications, such as catalysts,^5^ highly efficient stabilizers of photovoltaic panels,^6,7^ chemical-vapor deposition agents,^8^ pharmaceuticals for cancer and malaria treatments,^9,10^ and many others.^11,12^ At the same time, new features and properties of ferrocene, such as structural inconsistencies around the phase transition,^13^ the quasielastic-neutron-scattering spectra,^14^ or the presence of cooperative electric phenomena^15^ have been revealed, which are difficult to reconcile with the symmetry involving pseudo *D*_5_ axis^16,17^ associated with this molecule in the crystalline phases.

Soon after the discovery of ferrocene,^1,2^ when its unprecedented sandwich structure was proposed^18^ and verified by spectroscopy and X-ray diffraction,^18−22^ the molecular conformation, then described as the relative position of the Cp rings rotated about the molecular *D*_5_ axis, became the center of interest. Two conformations, staggered (antiprismatic, when the Cp rings are rotated one with respect to the other by torsion angle τ equal 36° about the molecular axis) and eclipsed (prismatic, for τ = 0°) were considered. The initial X-ray diffraction studies reported the staggered position of the Cp rings.^22^ In 1960, Willis, after being prompted by Edwards, Kington, and Mason, established by neutron diffraction that the Cp rings are disordered (Figure 1).^23−25^ Soon after, by employing electron diffraction, Bohn and Haaland^26^ found that the gaseous molecules equilibrate with the Cp rings eclipsed, while the staggered conformer is associated with the internal barrier of rotation of 3.8(13) kJ/mol.^27^ The high-resolution neutron diffraction study by Takusagawa and Koetzle^28^ confirmed the disorder of the Cp rings, with their partially occupied sites rotated from the staggered conformation by 9.0–15.5° about the molecular 5-fold axis (Figure 1). The disorder was hardly affected by the temperature between 298 and 173 K, but both static and dynamic disorder was considered.^29−33^ The disorder has been connected to the twinning and macroscopic properties of ferrocene crystals.^34^

**Figure 1:**
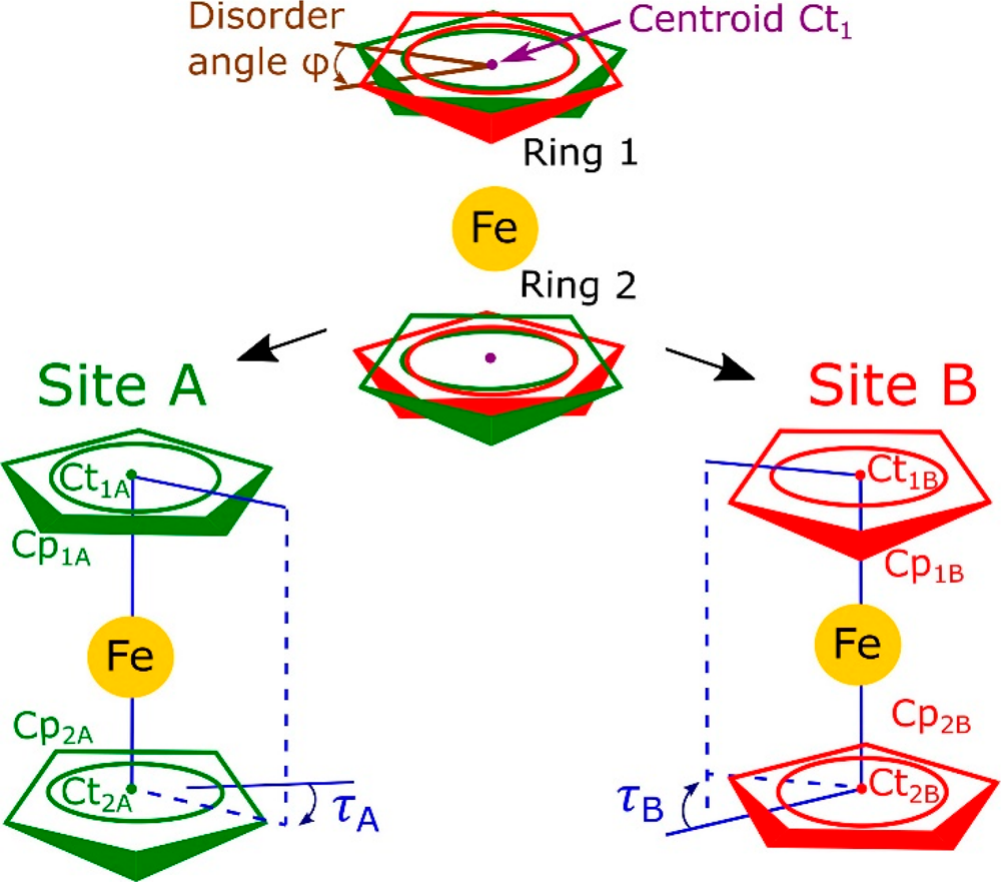
Schematic drawings of the ferrocene molecule in phase I (top) disordered in two sites A (green) and B (red). The scheme is idealized by superimposing the centroids of ring Cp_1_ sites Cp_1A_ and Cp_1B_ at one centroid Ct_1_ and the analogous one centroid Ct_2_ of ring Cp_2_ disordered in sites Cp_2A_ and Cp_2B_. The special position of Fe at the inversion center implies that Ct_1A_–Fe–Ct_2A_ and Ct_1B_–Fe–Ct_2B_ are linear, that pairs of ring sites Cp_1A_ with Cp_2A_ and Cp_1B_ with Cp_2B_ are parallel, and that torsion angles τ_A_ and τ_B_ are equal to 36°, corresponding to the staggered conformation of sites A and B (shown separately in the bottom drawings). Owing to the approximation of parallel ring sites Cp_1A_ and Cp_1B_, the disorder is described by one angle φ.

However, a low-temperature phase transition of ferrocene, marked by the heat-capacity anomaly at 164 K with a secondary peak at 169 K, was reported.^24,35^ The subsequent X-ray powder diffraction data confirmed the presence of a phase transition at 164 K.^36^ The triclinic (nonconventional) space group *F*1̅ with 16 molecules in the unit cell (*Z* = 16; for the conventional primitive unit cell, the *Z* number is 4) was assigned to this low-temperature phase, then labeled phase II.^37^ This result was corroborated by single-crystal X-ray diffraction.^38^ In this phase II, two symmetry-independent ordered molecules of ferrocene assume the *D*_5_-pseudosymmetric conformation, described either as rotated or twisted, where τ is about ±9°. A third low-temperature phase, labeled as phase III, of orthorhombic symmetry, space group *Pnma*, *Z* = 4, was also found.^39,40^ Its single-crystal structure determination revealed the *D*_5*h*_-pseudosymmetric eclipsed conformers, with their slightly bent Ct_1_–Fe–Ct_2_ axes (Ct_1_ denotes the centroid of ring Cp_1_ and Ct_2_ is the centroid of ring Cp_2_) lying on mirror planes.^41^ The structures of phases I and II are similar in the crystal packing and their lattice dimensions are related, but phase III is distinctly different in the packing of molecules, their site symmetry, and the ordered eclipsed molecular conformation (τ = 0°). The possible conformations of ferrocene molecules were associated with these three crystalline phases and compared to other molecules of metallocene compounds, for example, nickelocene,^42^ isostructural to ferrocene phase I, and ruthenocene, isostructural to ferrocene phase III.^43,44^ More recently, a new high-pressure ferrocene phase I′ was found above 3.2 GPa at 298 K,^45^ where the molecules are ordered in the staggered conformation due to the enhanced effect of the centrosymmetric crystal field. It was established later that phase I′ dominates the *p*–*T* phase diagram down to 90 K and up to 40 GPa at least.^46^ Phase I′ is the only structure, where the ferrocene molecules are present in the ordered staggered conformation of pseudo *D*_5*d*_ symmetry, which has been the most common graphical representation of ferrocene molecules and a frequent example of point groups in textbooks^16,47^ (also see https://en.wikipedia.org/wiki/Organometallic_chemistry), since the discovery of ferrocene, for decades before this ordered conformer was observed for the first time in 2013. It should be noted that in all crystalline phases, the FeCp_2_ molecules necessarily display some departures from the 5-fold symmetry. In the structure of ordered phase I′, the ring centroids and Fe dication (Ct_1_–Fe^2+^–Ct_2_) are exactly collinear and the Cp_1_ and Cp_2_ rings are exactly parallel in accordance with the *C*_i_ symmetry of the molecule; the departure from the 5-fold symmetry is only due to the Cp rings slightly nonperpendicular with respect to the molecular axis Ct_1_–Fe^2+^–Ct_2_. In phase I, the same applies to sites A and B separately, but their combined distribution is less symmetric due to the displacement of the rings and their inclination (Figure S4). In phases II and III the Cp rings are not quite parallel. In phase II at 101 K, the Cp planes are inclined by 0.46° and 0.76° for two independent molecules; the Ct_1_–Fe^2+^–Ct_2_ angles divert from 180° by 0.30° and 0.35°, respectively. In phase III at 98 K, the Cp_1_–Cp_2_ inclination and the diversion from linearity of Ct_1_–Fe^2+^–Ct_2_ are both equal to 0.52°. These angular distortions, inconsistent with the 5-fold symmetry, are relatively small, so it is not certain if they are inherent features of the ferrocene molecule or if they are induced by the crystal field. This is an important difference, because it results in a nonzero electric dipole moment of the ferrocene molecule.

When considering over 70 years of intense research on molecular and macroscopic properties of ferrocene, it is amazing that the early calorimetric, X-ray, and other studies overlooked the presence of yet another crystal form, now labeled by us as phase I″. On cooling ferrocene below 172.8 K, the average structure of phase I is preserved, but it becomes modulated in the new incommensurate phase I″. Below 163.5 K, phase I″ transforms into phase II. This previously unknown phase I″ is of key importance for ferrocene’s transformations and dielectric properties. Its structure–property relations are described below.

*Calorimetry.* A series of differential scanning calorimetry (DSC) measurements, performed with different rates of temperature changes on single-crystal and powdered ferrocene samples of different thermal history (Figure 2), illustrate the interplay of three transitions between four ferrocene phases I, I″, II, and III. These results are essentially in accordance with the previous calorimetric results,^24,25,39,40,48^ although we propose a revised interpretation including the presence of phase I″, previously neglected. On cooling from room temperature, all the samples display a subtle anomaly at 172.8 K, which was previously ascribed to an enigmatic subsidiary transition,^24^ while this is a characteristic anomaly associated with the second-order transition from monoclinic phase I to modulated phase I″. The continuous character of this transition is indicated by the anomaly shape and the lack of thermal hysteresis between the transition temperatures in the cooling (transition temperature defined by the anomaly onset) and heating (transition temperature defined by the anomaly minimum) runs, as shown in detail in Figure 2c. On further cooling, this small thermal anomaly is followed at 163.5 K by a strong sharp exothermal peak, characteristic of a first-order transition, when phase I″ transforms to triclinic phase II. For the single-crystal sample (runs A in Figure 2a), the successive transformations between phases I, I″, and II can be induced repeatedly by cycling the temperature up and down between 300 and 90 K. However, for the rough and fine powders (sample runs B and C), sometimes additional anomalies of strongly first-order type were observed in the temperature region between 100 and 120 K. In the previous reports these anomalies were ascribed to the disintegration of large crystallites^40,49^ but our study clearly shows that they mark the transitions of parts of the sample from phase II to phase III. This is evident from the reduced anomalies in the heating runs associated with the transitions between phases II, I″, and I. On heating, phase III does not undergo a reverse transition to phase II but transforms directly to phase I at 262 K, exhibiting a huge temperature hysteresis of about 150 K (*i*.*e*., from ca. 110 to 262 K). Although in our experiments we never fully converted the sample to phase III, the decrease in the thermal effect related to the transitions between phases II/I″/I in the heating run C allowed us to determine that 34% of the sample transformed to phase III. Considering this information, the transition entropies Δ*S*_II/III_ = 17.6(9) J mol^–1^ K^–1^ and Δ*S*_III/I_ = 16.1(8) J mol^–1^ K^–1^ were estimated. This latter value of Δ*S*_III/I_ is marginally lower than 17.13 J mol^–1^ K^–1^ previously obtained from the adiabatic calorimetry^39^ and than 16.6 J mol^–1^ K^–1^ from the DSC study.^40^ The entropy change associated with the transition from the triclinic phase II to the orthorhombic phase III was not determined before. The accumulation of entropy changes associated with transitions I/I″, I″/II (Figure 2d), and II/III gives the total entropy change Δ*S*_tot_ = 23.8(12) J mol^–1^ K^–1^, while the reverse transition immediately from phase III to phase I is associated with the substantially lower entropy gain. The entropy deficiency, estimated between 6.7 and 7.7 J mol^–1^ K^–1^, like the formation of phase III, is puzzling and requires further studies. In DSC runs B and C shown in Figure 2a, this phase was obtained as a result of the transformation of phase II. Alternatively, phase III can be grown by annealing the sample at about 200 K,^39,40^ but only after cooling it previously below 164 K to the triclinic phase II, as is clearly illustrated by the runs in Figure 2b. This experiment was performed for a fine-powdered sample, similar to that used for runs C in Figure 2a, but in this case no signs of transition to phase III during the cooling run to 95 K were detected. Nevertheless, the sample heated to 200 K and left for 15 h at this temperature exhibits the anomaly at 262 K characteristic of the transition between phases III and I. Although the mechanism of the transition to phase III is unclear, we believe that apart from numerous factors, such as internal strain, temperature gradient, and surface energy, the most crucial are microseeds of phase III, formed when the sample is cooled below 164 K. The heat of their formation contributes to the DSC baseline and to the peaks of the transitions to phases I″ and II, only. When temperature is elevated back to the range of phase I, around 200 K these microseeds gradually grow into larger regions of phase III during tens of hours at least. This slow transformation indicates that up to about 250–262 K phase III is thermodynamically more stable compared to phase I, but a substantial barrier separates the energy minima of phases I and III.

**Figure 2:**
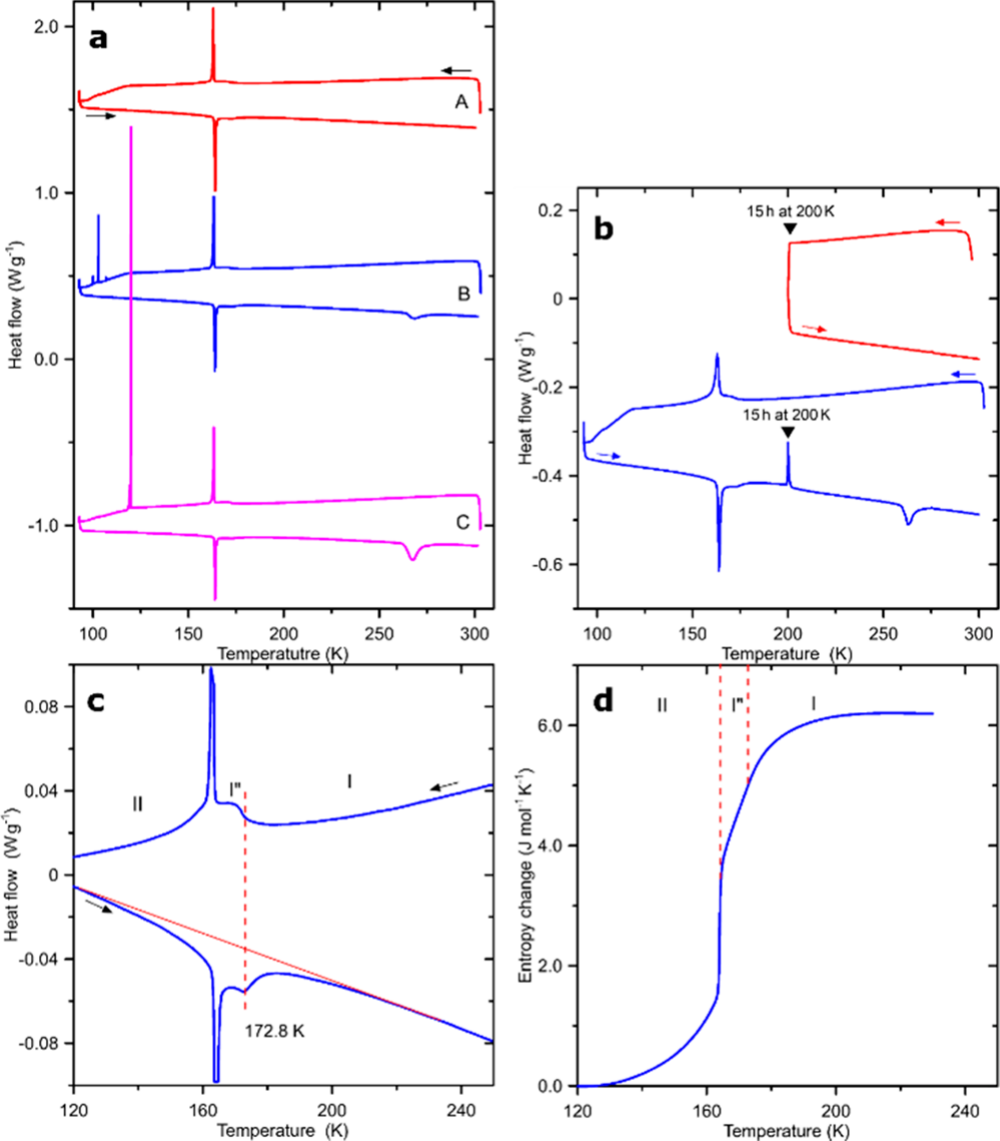
Sequences of DSC cooling and heating scans for ferrocene. (a) Three DSC consecutive cooling and heating runs for samples in the form of (A) a single crystal, (B) coarse powder, and (C) fine powder. For the single crystal A, the transition to phase III is absent, it occurs in 3 steps for coarse powder B, and it proceeds in one step for a large portion of fine powder C. (b) DSC runs for another fine-powder sample annealed at 200 K before (red) and after (blue) passing through the phase-transitions region. (c) Enhanced DSC signal for the cooling and heating runs in the temperature range of the transitions between phases I, I″, and II. (d) Entropy changes derived from the heating run for the single-crystal sample.

*Modulated Phase I″.* Our single-crystal X-ray diffraction data measured between 172.8 and 163.5 K contains satellite reflections indicating two modulation vectors **q**_1_ and **q**_2_ (Table 1), which do not change in all the temperature range of phase I″ (cf. Figures S1–S3). The average crystal structure of phase I″ approximates that of phase I; however, an incommensurate monoclinic space group in (3+2)d superspace is required for describing its symmetry (Table 1 and Tables S1 and S2).^50−52^ The modulation strongly affects the conformation, position, and interactions of the molecule in the crystal environment. The modulated structural changes are clearly different for the molecules disordered in two independent sites A and B, but their occupancy factors (SOF) remain equal to 0.5 within error. In phase I″, the description of conformations of disordered sites A and B and their relative positions become more complicated, due to the significant bending of the molecules, displacements of Cp-ring centroids and significant distortions from pseudo-*D*_5_ axis. For example, the bending angles ψ exceed 8° and cannot be neglected (Figure 3, Figure S4), and hence the approximations of angles φ for describing relative positions of ring sites A and B, or of torsion angles τ for the molecular conformation (Figure 1), become too broad. The molecular bending angle ψ and the displacements of centroids render the previously used torsion angles and rotations between disordered rings less adequate. A set of additional parameters describe the molecular tilts and displacements, including the displacements of the Fe atom off its averaged position at the inversion center, inclinations of rings sites Cp_1A_ to Cp_2A_ and Cp_1B_ to Cp_2B_, and distortions of linearity of ring centroids (Ct) with the Fe cation, Ct_1A_–Fe–Ct_2A_ and Ct_1B_–Fe–Ct_2B_. The disorder of ring Cp_1_ in two equally occupied sites A and B implies that apart from the Cp_1A_/Cp_2A_ and Cp_1B_/Cp_2B_ configurations, the molecular rings can be present in the “cross” positions, not related by symmetry operation (3) specified in Table 1. Such “cross” configurations Cp_1A_/Cp_2B_ and Cp_1B_/Cp_2A_ can be considerably populated, similarly to configurations Cp_1A_/Cp_2A_ and Cp_1B_/Cp_2B_. Consequently, the bending angles Ct_1A_–Fe–Ct_2B_ and Ct_1B_–Fe–Ct_2A_ have to be considered (Figure 3). In phase I″, angle Ct_1A_–Fe–Ct_2A_, changes between 171.4(4) and 180.0(4)° and angle Ct_1B_–Fe–Ct_2B_ between 171.6(4) and 179.9(5)°. Angles Ct_1A_–Fe–Ct_2B_ and Ct_1B_–Fe–Ct_2A_ are modulated in the same way between 173.5(5) an 178.4(5)°, but their modulation functions are shifted by 0.3*t*.

**Table 1:** Crystal Data of Incommensurate Phase I″ of Ferrocene at 166 K (for Detailed Information *cf*. Tables S1 and S2)

chemical formula, phase	Fe(C_5_H_5_)_2_, phase I″
crystal system	monoclinic
space group	*P*2_1_/*n*(αβγ)00(αβγ)00,^a^ 14.2.16.6^b^
wave vectors:	
**q**_1_	0.5000(8)a* + 0.3884(11)b* + 0.1665(13)c*
**q**_2_	–0.5000(8)a* + 0.3884(11)b* – 0.1665(13)c*
unit cell:	
*a*, *b*, *c* (Å)	5.7982(2), 7.5645(3), 8.9915(4)
β (deg)	92.911(3)
*V* (Å^3^)	393.86(3)
*Z*, density (g·cm^–3^)	2, 1.569

a Symmetry operations: (1) x1, x2, x3, x4, x5; (2) −x1 + 1/2, x2 + 1/2, −x3 + 1/2, x5 + 1/2, x4 + 1/2; (3) −x1, −x2, −x3, −x4, −x5; (4) x1 + 1/2, −x2 + 1/2, x3 + 1/2, −x5 + 1/2, −x4 + 1/2.

b https://stokes.byu.edu/iso/findssg.php

**Figure 3:**
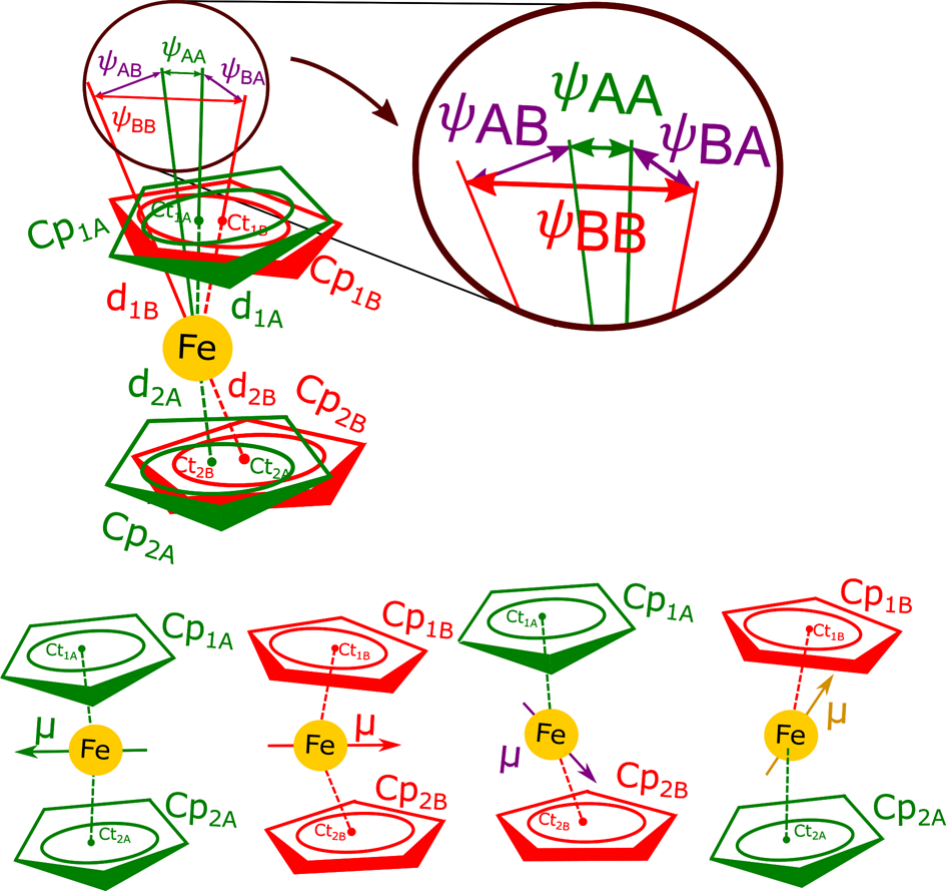
Conformational features of the ferrocene molecule in modulated phase I″ relevant to the electric dipole (μ) generation: (top) bending angles ψ_AA_, ψ_BB_, ψ_AB_ ,and ψ_BA_ supplementary of Ct_1A_–Fe–Ct_2A_, Ct_1B_–Fe–Ct_2B_, Ct_1A_–Fe–Ct_2B_, and Ct_1B_–Fe–Ct_2A_ to linearity, respectively, as well as distances (d) Ct_1A_–Fe, Ct_1B_–Fe, Ct_2A_–Fe, and Ct_2B_–Fe in disordered sites A (green) and B (red); (bottom) possible configurations AA, BB, AB, and BA assumed in the structure. All bending angles ψ, distances *d*, Fe^2+^ shifts from the inversion center, relative rotations between disordered Cp rings (φ), and conformational torsions between rings 1 and 2 are modulated.

In the further discussion, we will focus on the positional modulations contributing to the molecular dipole moment, for example the distortions from the Ct_1_–Fe–Ct_2_ linearity, Ct–Fe distances, and Fe displacements. The conformational parameters can be plotted as a function of [*t*,*u*] coordinates for phase I″. The molecular structure observed in phase I″ provides the new information about the structure–property relations of all ferrocene compounds.

Figure 4 shows some “snapshots” of the structure, but a clear visual illustration of the modulation is provided by the animations in Videos S1 and S2. The relative positions of disordered sites are modulated, as illustrated in the plots in Figures S5 and S6.

**Figure 4:**
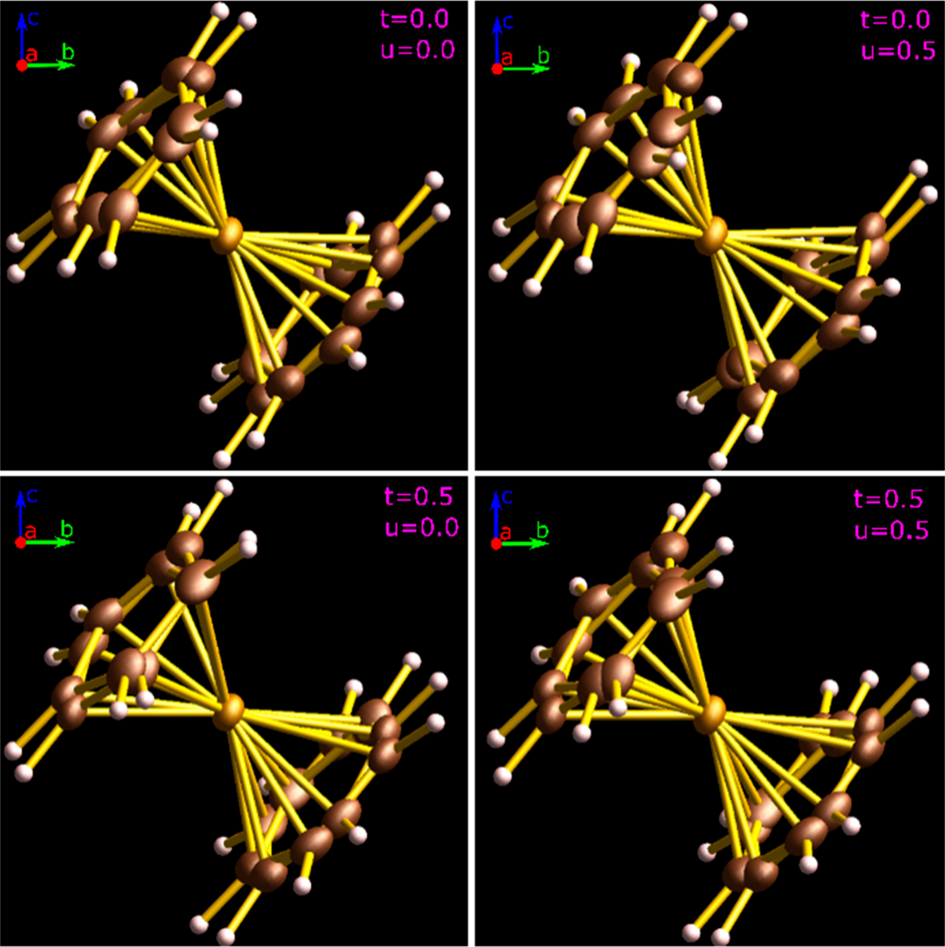
Snapshots of disordered ferrocene molecule in phase I″ viewed along the crystal direction *a*, at the modulation *t*-coordinates 0 and 0.5 (left to right) and *u*-coordinates 0 and 0.5 (top to bottom). Videos S1 and S2 show the animation of conformational and positional changes along the modulated space vectors. The atoms of Cp rings, disordered in two equally occupied sites, are plotted at the 30% probability level.

The animations illustrate, apart from the conformational features of the disordered molecules, the displacements and librations of sites A and B along the modulation coordinates [*t*,*u*]. The (3+2)d superspace of the structure in phase I″ requires either that selected [*t*,*u*] coordinates be chosen for the structural drawings (as in Figure 4) or that changes of structural parameters be mapped in the [*t*,*u*] coordinates, as is done in Figure 5 (*cf*. Figure S7). For describing the conformational changes, because centroids Ct_A_ and Ct_B_ do not superimpose, we have chosen the inclinations of C1–H1–Fe planes for sites A and B, respectively. They illustrate that the amplitude of conformational changes of the molecule in site A, of over 40°, is considerably larger than that in site B, of over 27.5° (Figure 5).

**Figure 5:**
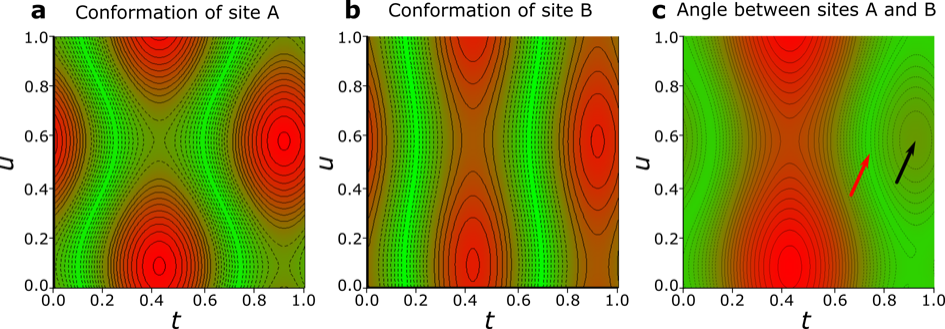
Modulation map in [*t*,*u*] coordinates of molecular conformation, measured by dihedral angles between planes of atoms: (a) C1_1A_–H1_1A_–Fe and C1_2A_–H1_2A_–Fe, the minimum angle is 0.8(5)°, average 20.1(5)° and maximum 42.4(5)°; (b) C1_1B_–H1_1B_–Fe and C1_2B_–H1_2B_–Fe, angles min. 0.4(7)°, average 15.8(11)°, and max. 28.1(20)°; and (c) C1_1A_–H1_1A_–Fe and C1_2B_–H1_2B_–Fe, angles min. 1.3(12)°, average 22.1(11)°, and max. 53.2(3)°. The full and dashed contours, 2° per line, mark the levels above and below the average value, also highlighted by the red (above) and green (below) background. In panel (c), there is a local maximum of 16.0(5)° indicated by the black arrow; the total minimum is indicated by the red arrow.

In ferrocene phase I, the disorder was usually described by the angular displacement φ, measured between the corresponding atoms of the Cp ring in sites A and B: C_iA_–Cp_c_–C_iB_, where indices *i* = 1–5 label atoms, letters A and B label the disordered sites of the Cp ring, and Cp_c_ is the centroid common for rings A and B. However, so defined angle φ provides only an approximate description of the disorder, due to the distortions of the molecular conformation from the *D*_5_ symmetry and because the centroids of rings A and B do not coincide. It can be clearly seen from Figure 5 and Videos S1 and S2 that the pseudo-*D*_5_ axes of sites A and B are displaced in precession-like positions and that the Cp rings associated with these sites are not parallel. Consequently, each of the Cp-ring disordered sites A and B has its individual centroid. Moreover, the Fe atom is also displaced from its average position at the inversion center. Therefore, instead of using the Cp ring centroid in the calculations, we have described the molecular conformation and the relative position of the disordered sites A and B with dihedral angles between planes defined by atoms Fe–C_1A_–H_1A_ and Fe–C_2B_–H_2B_ (*i* = 1, 2 for rings 1 and 2, respectively); for the ideally collinear *D*_5_ axes for sites A and B they are equivalent to torsion angles involving one common centroid (*cf*. Figure 1).

The positions of molecules in sites A and B can be associated with the intermolecular interactions in the crystal field, which is also manifested in the modulated position of the dication Fe^2+^ about its averaged position at the inversion center. Its displacements along crystal directions [*x*], [*y*], and [*z*] are mapped in Figure 6. The smallest Fe displacements are along the [*x*] direction, and much larger displacements are along [*y*] and [*z*]. They correspond with the directions of the strongest interactions between molecules in the crystal structure. The Fe^2+^ dication is strongest displaced along the plane of hydrogen bonds CH···π, lying approximately along directions [011] and [011̅]. Moreover, directions *b* and *c* display the strongest anomalous linear compression, when ferrocene phase I transforms into phase I′ at 3.2 GPa, while no anomalous compression was detected along the *a* direction, where no CH···π bonds exist.^45,46^ The CH···π bonds are considered to be the strongest intermolecular interactions in the crystal structure of ferrocene I.^43,45^ Therefore, the elimination of disorder of the Cp rings carrying both the H-donor and H-acceptor groups at 3.2 GPa is reflected in the anisotropic anomalous compression.

**Figure 6:**
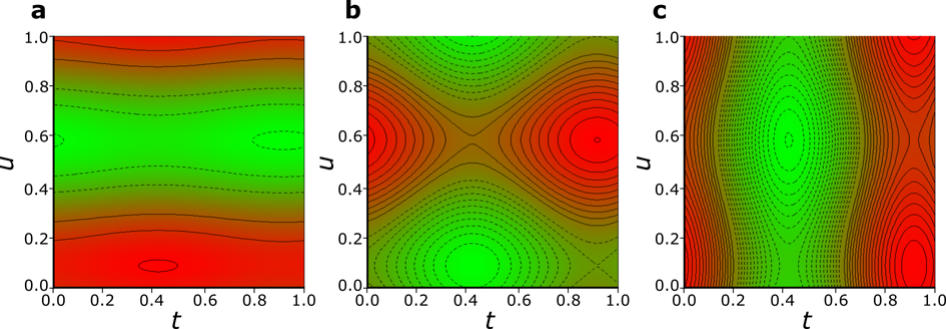
[*t*,*u*]-Modulation map of Fe atom displacement from the average position at the inversion center along crystal directions (a) [*x*], (b) [*y*], and (c) [*z*]. The full and dashed contours mark the excess and deficient steps 0.005 Å per line, respectively, from the average of position at the inversion center. The Fe displacements are modulated within −0.0152 ≤ *x* ≤ +0.0152(5), −0.0547 ≤ *y* ≤ +0.0547(5), and −0.0941 ≤ *z* ≤ +0.0941(5) Å.

The Fe^2+^ displacements are coupled with other modulation types in the structure of phase I″. Another molecular feature of ferrocene phase I″ is significantly nonparallel Cp rings. Their inclinations within and between sites A and B are modulated off the parallel positions: the inclination-angle amplitude is modulated between 0.04° and 2.82° for rings Cp_1A_ and Cp_2A_ and between 0.03° and 3.33° for site B, as illustrated in Figure 7. This suggests that nonparallel positions of the Cp rings are favored. In the triclinic ferrocene phase II at 101 K, the Cp rings are inclined by 0.76° and 0.46° for two symmetry-independent molecules;^38^ in the orthorhombic phase III at 98 K, this dihedral angle is 0.46°. Also in ruthenocene phase α, isostructural with ferrocene phase III, the dihedral angle is 0.50°,^53−55^ whereas in the high-pressure phase β the dihedral angle assumes 1.4° and increases up to 2.5° when an anagostic hydrogen bond CH···Ru is induced in the compressed structure.^56^ It is characteristic that the modulations of the inclined rings are correlated between sites A and B, which confirms their coupling to the crystal environment.

**Figure 7:**
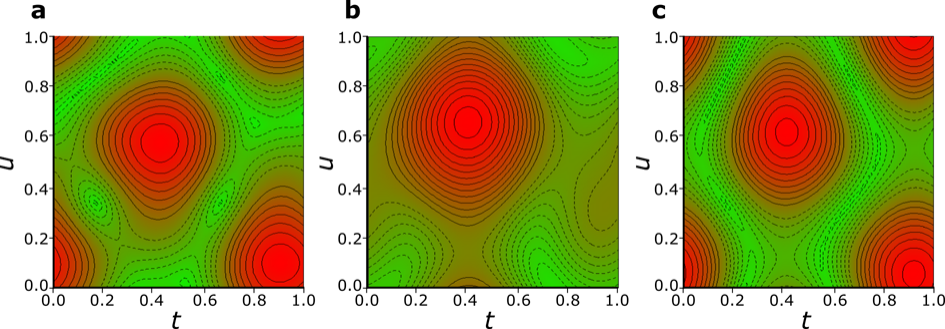
Modulation [*t*,*u*]-map of inclinations between half-occupied disordered rings: (a) Cp_1A_ and Cp_2A_, with the minimum angle 0.0(5)° (green), average 1.3(4)°, maximum 2.8(4)° (red); (b) Cp_1A_ and Cp_1B_, the angle min. 0.9(4), average 2.9(4)°, and max. 5.6(4)°; (c) Cp_1B_ and Cp_2B_, the angle min. 0.03(45)°, average 1.5(4)°, and max. 3.3(4)°. The full and dashed contours mark the excess (red background) and deficient (green background) steps of 0.20°.

The displacements of the Fe^2+^ cation and the inclinations of Cp-rings are connected with the molecular distortions, which generate the molecular dipole vector **μ**. The bending angle ψ and distances *d*_1_ and *d*_2_ between Fe and Cp-ring centroids Ct (Figure 3) are connected to the horizontal (perpendicular to the molecular axis) and vertical (along the molecular axis) **μ** components μ_h_ and μ_v_, respectively. As expected, the changes of Cp–Fe distances *d*_1_ and *d*_2_ are much smaller compared to the displacements due to the Ct–Fe–Ct angle bending (Figures S8 and S9), hence the nearly horizontal orientation of the dipole moments. The offset of charges Fe^2+^ and two Cp^–^ to the opposite sides of the molecular center generates a dipole moment. The horizontal component μ_h_ is proportional to the sinus of the bending angle, ψ, and the vertical component μ_v_ is proportional to the (*d*_1_ – *d*_2_)/2 difference. But most importantly, the value of the dipole moment, its direction, and sense depend on the sites assumed in the disordered molecule by the Cp rings (Figure 8 and Videos S3, S4, and S5). One molecule can assume one of the possible configurations of its disordered Cp rings: Ct_1A_–Fe–Ct_2A_, Ct_1B_–Fe–Cp_2B_, Cp_1A_–Fe–Cp_2B_, and Cp_1B_–Fe–Cp_2A_ (respectively, conformations AA, BB, AB, and BA, as indicated in Figure 3). If we excluded the cross-term configurations, AB and BA, each molecular site could be associated with the probability of 0.5 with configuration AA or BB, and hence either with dipole **μ**_AA_ or **μ**_BB_. The amplitudes of their modulations are |**μ**_AA_| = 3.97 × 10^–30^ C·m (1.19 D) and |**μ**_BB_| = 3.87 × 10^–30^ C·m (1.16 D). Figure 8 and Videos S3, S4, and S5 show that dipoles **μ**_AA_ and **μ**_BB_ are nearly antiparallel and librate one about another along the modulation. The presence of configurations AB and BA cannot be excluded, and they would decrease the populations of configurations AA and BB, so that the summed probabilities of all configurations is 1. The amplitudes of the mixed-term modulations **μ**_AB_ and **μ**_BA_ are 3.01 × 10^–30^ C·m (0.90 D). It can be observed in Figure 8 and Videos S3, S4, and S5 that they are nearly parallel. As expected, the μ_v_ components are small due to the high stiffness of Fe–C bonds,^46^ and all dipole vectors **μ**_AA_, **μ**_BB_, **μ**_AB_, and **μ**_BB_ are directed close to the horizontal plane.

**Figure 8:**
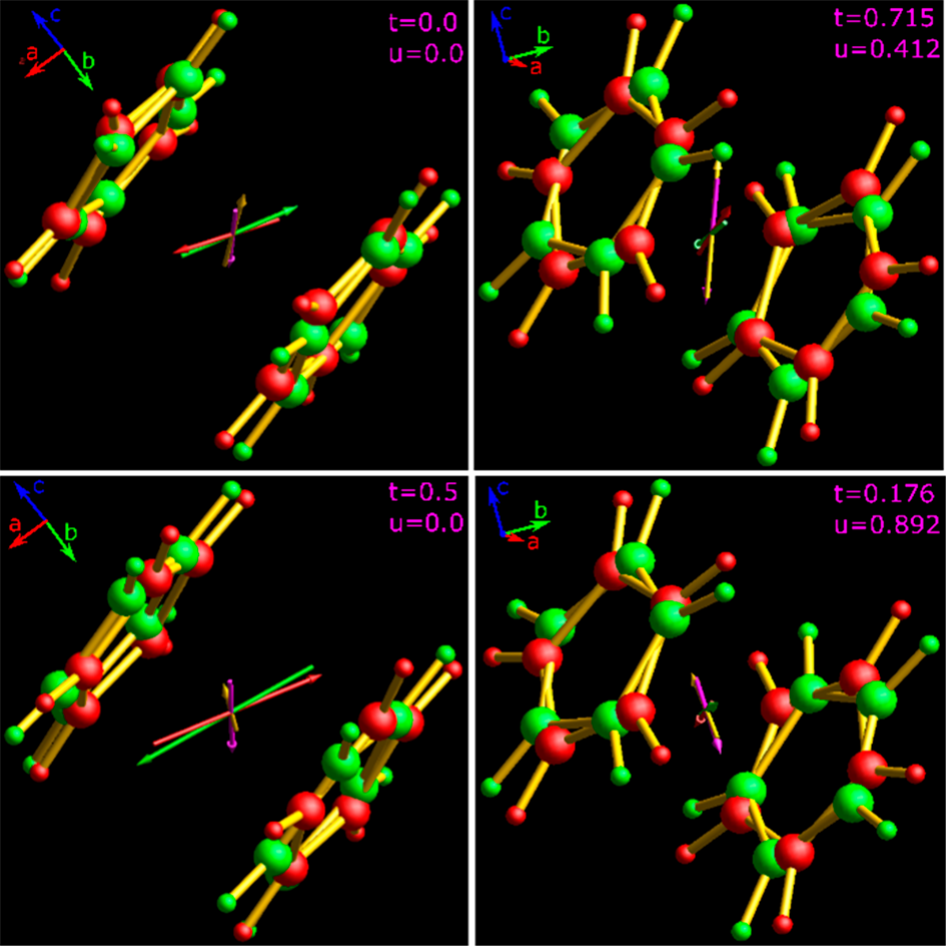
Dipole moments of a ferrocene molecule in phase I″ of configurations AA, BB, AB, and BA, shown as green, red, purple, and yellow arrows, respectively, at selected [*t*,*u*] coordinates. The maximum lengths of these modulated dipoles are 1.30, 1.27, 1.04, and 1.04 D; atoms disordered in site A are green, and those in site B are red (*cf*. Videos S3, S4, and S5 and Figure S12).

Both dipole moments **μ**_AA_ and **μ**_BB_ reverse their sense along the modulation coordinates, apart from their librations, which are much lower in amplitude due to the modulation of components μ_v_ (Figures S10 and S11). For the cross-site dipole moments, **μ**_AB_ and **μ**_BA_, their vector sense is not changed, and the magnitudes of the vectors are not modulated, either; these dipoles only perform a librational motion. Dipoles **μ**_AA_ and **μ**_BB_ are approximately perpendicular to dipoles **μ**_AB_ and **μ**_BA_.

The magnitudes of molecular dipole moments revealed in ferrocene phase I″ are much larger compared to those in other ordered phases: in phase I′ the dipole is zero (by symmetry); in phase II two independent molecules have dipoles 0.056 and 0.046 D; and in phase III, μ is 0.072 D. These values well agree with those calculated by Bermúdez-García.^15^

*Dielectric Spectroscopy.* In the frequency range studied between 1 kHz and 5 MHz the dielectric response of polycrystalline ferrocene is characterized by low dielectric losses (tangent of dielectric loss <0.003) and low frequency dependence. Hence only the real part of the electric permittivity measured at 100 kHz is plotted as a function of temperature in Figure 9. On cooling, in monoclinic phase I, the electric permittivity increases progressively up to the transition to modulated phase I″ at 172.8 K. This indicates that an additional contribution to the dielectric response of ferrocene occurs, such as fluctuations of electric dipole moments or fluctuations of charge distribution. In phase I, the ferrocene molecules are centrosymmetric and, therefore, should be devoid of electric dipoles. However, this symmetry is broken in modulated phase I″, where the molecules bend and acquire electric dipoles of values differentiated along the modulation vectors. It is evident that locally incommensurate domains form already well above the transition point, in the temperature range of disordered phase I. Such dynamic domains can be created and annihilated by solitons, inducing fluctuations of electric dipole moments.^57^ The density of incommensurate regions grows when lowering the temperature, and eventually, all the structure becomes modulated. In phase I″ and partly also in the commensurate ordered phase II, the dynamics of dipolar fluctuations is progressively suppressed, as can be inferred from the decreasing value of the electric permittivity. The dielectric response of ferrocene described here is fully consistent with calorimetric data. In Figure 2c, the transition between phases I and I″ is preceded by a large DSC signal stretching between ca. 220 K and the transition point. This pretransitional effect originates from critical fluctuations involving creating and disappearing incommensurate areas within commensurate phase I. Also, the residual dynamics observed in the dielectric response in phase II is reflected as a low-temperature tail of the first-order transition between phases I″ and II.

**Figure 9:**
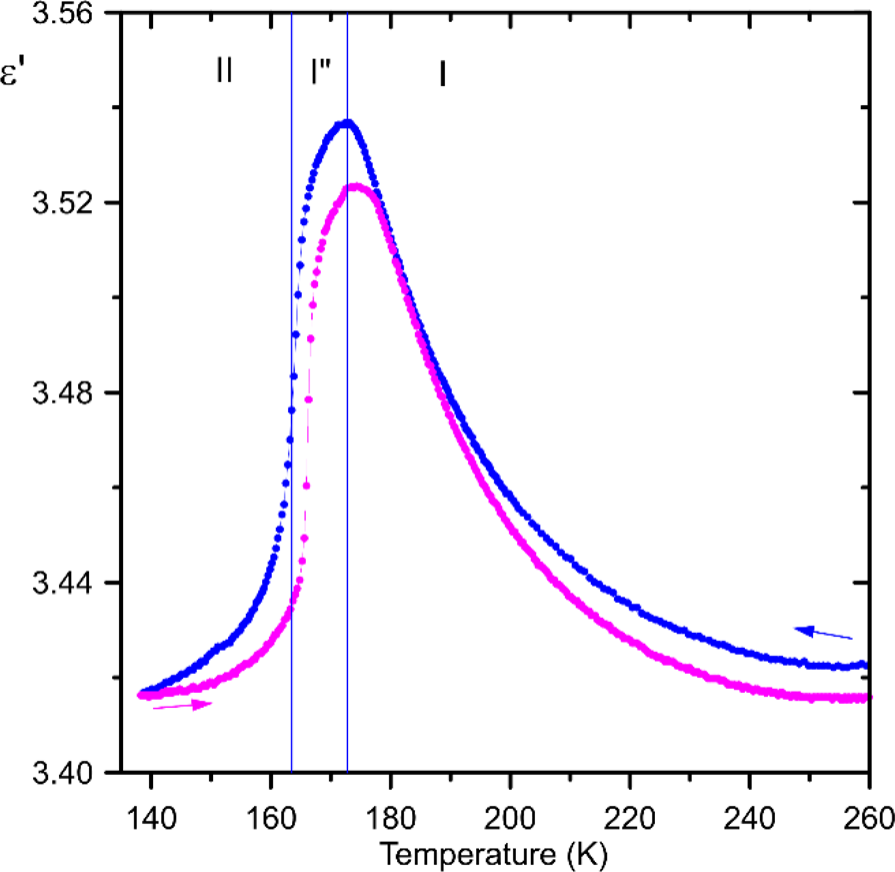
Real part of electric permittivity of ferrocene measured on cooling (blue) and heating (pink) of the pellet in the region of transitions between phases I, I″, and II. The blue vertical lines mark the transition temperatures determined from the DSC cooling run.

Thus, the dipole moment of the ferrocene molecule appears to be an intrinsic feature, which can be stabilized in low-temperature phases II and III. At elevated temperatures, the molecular bending disappears due to the coupling of quick molecular vibrations with the lattice-mode vibrations in phase I. The thermally activated vibrations of the central dication also persist in the high-pressure phase I′, where the crystal field plays a predominant role for the centrosymmetric molecular sites. When phase I is cooled from room temperature, the fast rotavibrations of molecules are gradually dumped, leading to momentary conformational changes increasing the dipole moments and local dipolar fluctuations generated in soliton waves and around structural defects. On approaching phase I″, the population of such dipole moments and their changes increases, as indicated by the anomalous dielectric response.

The modulated phase I″ revealed in this study clarifies the origin of the “secondary” peak in the calorimetric studies on ferrocene, the dielectric response of ferrocene and the presence of anomalous thermal expansion of the crystal about 170 K. Owing to the modulation of phase I″, detailed structural information about the conformation and disorder of molecules can be derived. Both the disorder and the conformational changes of each disordered site involve the Fe^2+^ displacements from its central position, the inclinations of Cp rings and the molecular bending, leading to a considerable dipole moment of the molecule. These results show that the dipole moment is an intrinsic feature of the ferrocene molecule, which is highly adaptive to the environment through the exceptional flexibility involving conformational transformations, molecular bending, disordering, and switching between p-orbital configurations. This conformational flexibility is reflected in the bent structures of ferrocene derivatives with substituted Cp rings.^58^ Due to the bent conformation, the molecular symmetry of unsubstituted FeCp_2_ can markedly depart from the 5-fold axis. However, the bending becomes averaged for thermally activated rotations in ferrocene phase I and other isostructural metallocenes, for example, in vanadocene and nickelocene.^59^ The obtained structural information complements the *p*–*T* phase diagram of ferrocene. It explains the puzzling circular route of its transformations in the cooling and heating runs: on cooling, the disordered phase I at 172.8 K transforms to the modulated phase I″, which precedes the transition at 163.5 K to the metastable triclinic phase II. Phases I, I″, and II preserve the network of hydrogen bonds CH···π favoring the staggered conformation. The slow kinetic process of the transitions between phases II and III is due to the structural reconstruction involving intermolecular interactions and conformational transformations of molecules. The flexibility of the FeCp_2_ molecule—involving Cp-rings rotations, Cp-Fe-Cp bending, and the connected dipole moment changes—are consistent with the adsorption properties of ferrocene,^60^ its interactions with solvents,^61^ and the dielectric response,^15^ which can be applicable to other metallocene compounds. Ferrocene phase I″ is the first modulated crystal structure revealed for metallocenes so far. Hence the consequences of the existence of phase I″, and in particular for the transition for otherwise unique ferrocene phase II, still remain to be investigated and understood, in connection with the physical and chemical properties of metallocenes in general. Presently, the phase diagram of ferrocene includes five phases (I, I′, I″, II, and III) and it is remarkable that two phases of this prototypical metallocene were revealed during the past decade. The occurrence of this first incommensurate phase among sandwich compounds provides a new type of conformational modulation for the already impressive list of modulated compounds.^62−67^

## Supporting Information Available

Experimental details on calorimetry, electric permittivity measurements, X-ray diffraction, molecular distortions breaking the *C*_5_, labels of the Cp rings centroids, references; *unwrap* of the scXRD diffraction CCD images (Figures S1–S3); conformations of modulated phase I″ (Figure S4); modulation maps (Figures S5–S11); snapshots of the ferrocene molecules in phase I″ (Figure S12); tables of crystal data and structural parameters (PDF)
Animation of the ferrocene molecule in phase I″ (AVI)
Animation of the ferrocene molecule in phase I″ (AVI)
Animation of the dipole moments of disordered sites of ferrocene molecule in phase I″ (AVI)
Animation of the dipole moments of disordered sites of ferrocene molecule in phase I″ (AVI)
Animation of the dipole moments of disordered sites of ferrocene molecule in phase I″ (AVI)
Transparent Peer Review report available (PDF)

## Supplementary Material

jz3c00215_si_001.pdf

jz3c00215_si_002.avi

jz3c00215_si_003.avi

jz3c00215_si_004.avi

jz3c00215_si_005.avi

jz3c00215_si_006.avi

## ABBREVIATIONS

Cp: cyclopentadienyl anion
Ct: Cp-ring centroids
*p*: pressure
*T*: temperature
μ: dipole moment
ψ: bending angle
φ: rotations between disordered Cp rings
τ: conformational torsions angles
