# Supplementary material for: Dipole-Moment Modulation in New Incommensurate Ferrocene
Source: J Phys Chem Lett. 2023 Mar 23;14(13):3111–9. doi: 10.1021/acs.jpclett.3c00215 (PMC10084461; doi:10.1021/acs.jpclett.3c00215)
Supplement: Supplementary file 1 — jz3c00215_si_001.pdf [file jz3c00215_si_001.pdf]

Supporting Information

Dipole-Moment Modulation in New Incommensurate Ferrocene

Andrzej Katrusiak[a*], Michalina Rusek[a], Michal Dušek[b], Václav Petříček[b] and Marek Szafrański[c]

[a] Faculty of Chemistry, Adam Mickiewicz University, Uniwersytetu Poznańskiego 8, 61-614 Poznań

[b] Institute of Physics, Czech Academy of Sciences, Na Slovance 2, 182 21 Praha

[c] Faculty of Physics, Adam Mickiewicz University, Uniwersytetu Poznańskiego 2, 61-614 Poznań

*Email: katran@amu.edu.pl

Table of Contents

Experimental: S2
Calorimetry S2
Electric permittivity measurements S2
X-Ray Diffraction S2
Molecular distortions breaking the C_5 .... S3
Labels of the Cp rings centroids (Ct): S3
References S3
Figure S1: Section (1/2 *k l*) of ferrocene phase I'' at 166 K S4
Figure S2: Section (-1/2 *k l*) of ferrocene phase I'' at 166 K S5
Figure S3: Section (3/2 *k l*) of ferrocene phase I'' at 166 K S6
Figure S4: Conformations of ferrocene molecule in modulated phase I'' S7
Figure S5: The [*t*,*u*]-modulation map of angle $C1_{1A}$—Fe—$C1_{1B}$ S8
Figure S6: The [*t*,*u*]-modulation map of distance $C1_{1A}$—$C1_{1B}$ S9
Figure S7: The [*t*,*u*]-modulation map of angles C—C—C S10
Figure S8: Ring-centroid A to Fe dication distances S11
Figure S9: Ring-centroid B to Fe dication distances S11
Figure S10: Bending of the molecular axis for sites AA and BB S12
Figure S11: Bending of the molecular axis for sites AB and BA S12
Figure S12: Selected snapshots of the $FeCp_2$ molecule in the phase I'' S13
Table S1: Crystallographic data of ferrocene phase I'' at 166 K/ 0.1 MPa S14
Table S2: Structural parameters of ferrocene phase I'' at 166 K/0.1 MPa S15
Video S1: Animation of the ferrocene molecule in phase I'' perpendicular to pseudo-D_5 axis S16
Video S2: Animation of the ferrocene molecule in phase I'' along the pseudo-D_5 axis S16
Video S3: Side view of dipole moments in ferrocene phase I'' S16
Video S4: Dipole moments in ferrocene phase I'' along the pseudo-D_5 axis S16
Video S5: Dipole moments in ferrocene phase I'' viewed perpendicular to the pseudo-D_5 axis S16

Experimental

Calorimetry. Differential scanning calorimetry (DSC) measurements on single-crystal and powdered ferrocene samples were carried out with a Q2000 calorimeter (TA Instruments); the samples sealed in aluminium cans were cooled/heated at a rate of 10, 3 and 0.3 K/min. Indium was used for the temperature and enthalpy calibration, and synthetic sapphire was used for the specific heat calibration.

Electric permittivity measurements. Relative complex electric permittivity $\varepsilon = \varepsilon' - i\varepsilon''$ was studied on pressed pellets with silver electrodes deposited on the parallel surfaces. The pellets were 13 mm in diameter, 0.50 mm thick. The dielectric functions were measured in the frequency range from 1 kHz to 5 MHz with a Hewlett-Packard 4192A impedance analyser. The amplitude of the ac measuring electric field was about 4 V/cm. The rate of temperature change was 0.3 K/min in the phase-transitions regions and 0.7 K/min outside the transitions.

X-Ray Diffraction. The single-crystal X-ray diffraction was measured as a function of temperature on a four-circle Oxford-Diffraction diffractometer equipped with an EOS CCD detector and Oxford Cryostream attachment. The temperature was calibrated with the precision of 0.1 K. The high-quality single crystals were carefully selected and the experiments were performed only in the cooling runs, in order to avoid the crystal cracks on the transition to the triclinic phase, which could considerably deteriorate the quality of the diffraction data. Several samples were used to ensure the repeatability the results and elimination of errors.
The data were processed by CrysAlis^{Pro}, the initial structure found by Superflip[1], and the modulated structure refined, analyzed and graphically illustrated by Jana2020 – the not yet published successor of Jana2006[2]. For the data set at 166 K, the refined *q*-vectors were [0.4997(11), 0.3880(14), 0.1675(16)] and [-0.5002(12), 0.3887(17), -0.1654(19)]. Hence q_1=[0.500, 0.3884, 0.166] and q_2=[-0.500, 0.3884, -0.166]. It was evident that the α components are ± 0.5, the β components are equal, and the γ components are equal with opposite signs, making possible the description either as an (3+2)-D modulated structure or an (3+1)-D-modulated twinned structure. The absence of the cross satellites, arose as a combination of both *q*-vectors, favoured the description as a twinned structure; however, such a model could not be satisfactorily refined. Therefore, we described the structure as a (3+2) dimensional one, assuming that the cross satellites were not detected because of their weakness.
The superspace symmetry was determined by examining the systematic absences, as No 14.2.16.6[3], applied after a transformation to the standard setting with the monoclinic angle β and average space group $P2_1/n$. It should be noted that although the α components are ± 0.5, these values are not dictated by the symmetry. Therefore, no super-centred setting was necessary.
Structure solution by Superflip returns, for the case of a (3+2)-D structure, only the average structure. The refined average structure contained large atomic displacement parameters (ADP) for carbon atoms indicating that the ferrocene ring is strongly modulated. Modulation waves were refined from small starting values, leading to the structural model with one position and one ADP wave for each wave vector $\boldsymbol{q}_1$ and $\boldsymbol{q}_2$. This structure model had a good fit of both main reflections and satellites; however, the ADP parameters of carbon atoms were still large, indicating the structure contains two split modulated positions of the Cp ring. We

modelled the two split positions by two 0.5:0.5 occupied rings undergoing the rigid-body modulation displacements[4]. The ADPs of the ferrocene rings and their modulations were described by TLS tensors[5]. We verified the rigidity assumption by comparing it with a structure model composed of individual atoms. For such a model, many more structural parameters were refined, but reliability factors *R* and *S* remained similar to those of the rigid-body model. We also verified the assumption that the molecular positions are split and that their description with only one molecule with large ADPs is not sufficient: such a description led to high *R* values (0.045 and 0.109 for main reflections and satellites, respectively) and pronounced difference Fourier maxima along the Cp ring. The final crystallographic and experimental data are summarized in Table S1; the average-structure ferrocene phase I'' data has been deposited with the Cambridge Structural Database (CCDC No. 2216305); full refinements files with modulation parameters have been deposited in the Bilbao Incommensurate Crystal Structure Database (B-IncStrDB), under record number Fxhs6DE1VaT. The full set of JANA files can be obtained from the authors (A.K. and M.D.).

Molecular distortions breaking the C_5 symmetry
Displacements of cation Fe^{2+} with respect to the anionic Cp rings, resulting in the dipole-moment modulation in the incommensurate ferrocene phase I''

Labels of the Cp rings centroids (Ct):

Centroid of the Cp ring disordered in site A:	Ct_{1A}
Centroid of the Cp ring disordered in site A transformed through #s3t1,0,1:	Ct_{2A}
Centroid of the Cp ring disordered in site B	Ct_{1B}
Centroid of the Cp ring disordered in site B transformed through #s3t1,0,1:	Ct_{2B}

References

(1) Palatinus, L.; Chapuis, G. SUPERFLIP - A Computer Program for the Solution of Crystal Structures by Charge Flipping in Arbitrary Dimensions. *J. Appl. Crystallogr.* **2007**, *40* (4), 786–790. https://doi.org/10.1107/S0021889807029238.

(2) Petrícek, V.; Dušek, M.; Palatinus, L. Crystallographic Computing System JANA2006: General Features. *Zeitschrift fur Krist.* **2014**, *229* (5), 345–352. https://doi.org/10.1515/zkri-2014-1737.

(3) Stokes, H. T.; Campbell, B. J.; Van Smaalen, S. Generation of (3 + d)-Dimensional Superspace Groups for Describing the Symmetry of Modulated Crystalline Structures. *Acta Crystallogr. Sect. A Found. Crystallogr.* **2011**, *67* (1), 45–55. https://doi.org/10.1107/S0108767310042297.

(4) Petricek, V.; Coppens, P.; Becker, P. Structure Analysis of Displacively Modulated Molecular Crystals. *Acta Crystallogr. Sect. A* **1985**, *41* (5), 478–483. https://doi.org/10.1107/S0108767385001027.

(5) Schomaker, V.; Trueblood, K. N. On the Rigid-Body Motion of Molecules in Crystals. *Acta Crystallogr. Sect. B Struct. Crystallogr. Cryst. Chem.* **1968**, *24* (1), 63–76. https://doi.org/10.1107/s0567740868001718.

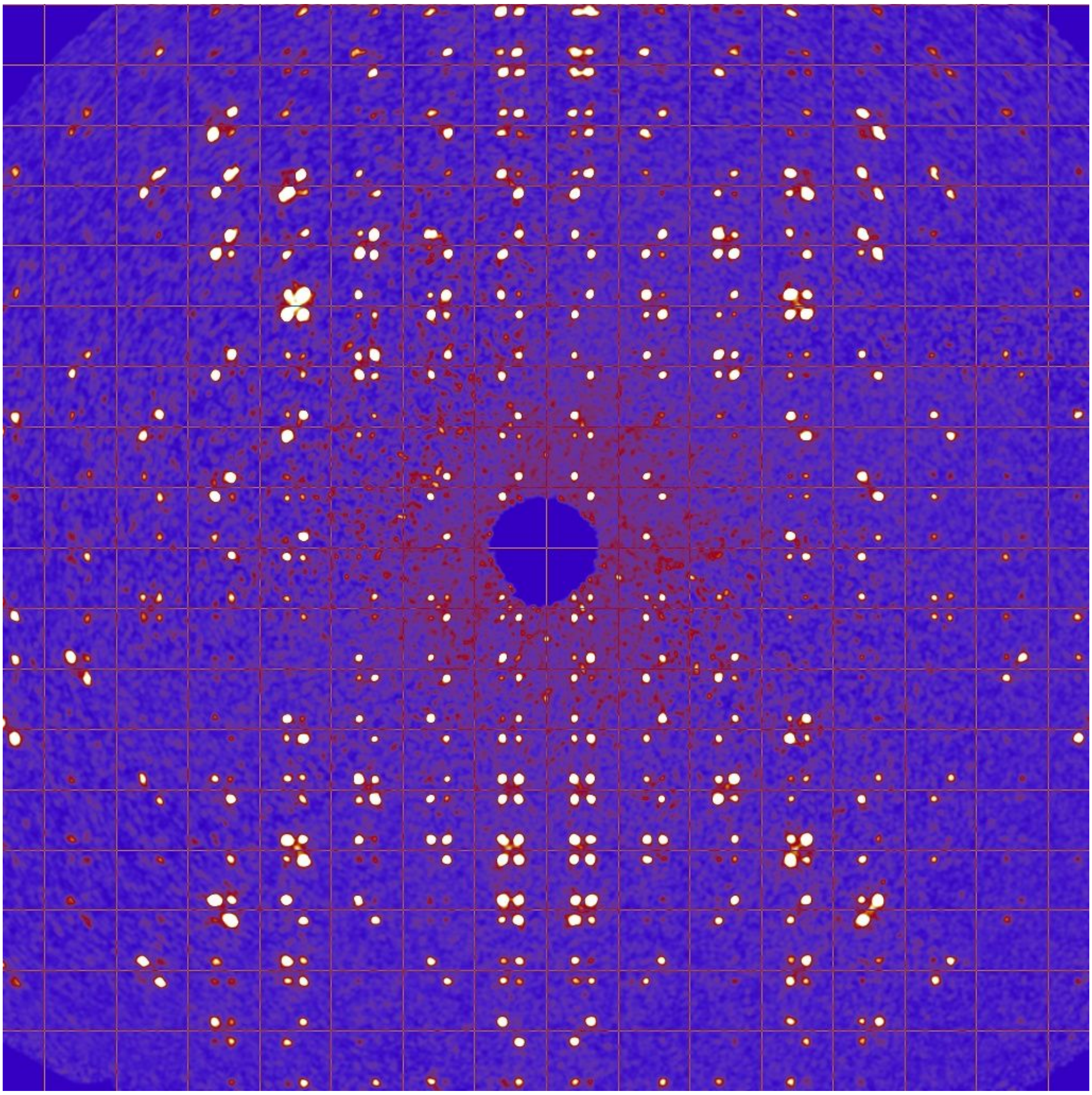

Figure S1. **Section (1/2 *k l*) of ferrocene phase I'' at 166 K**, calculated from CCD images by CrysAlis^{PRO} instruction *unwrap* of the scXRD diffraction CCD images. The *unwrap* integration range from 0.4*a** to 0.6*a** was applied.

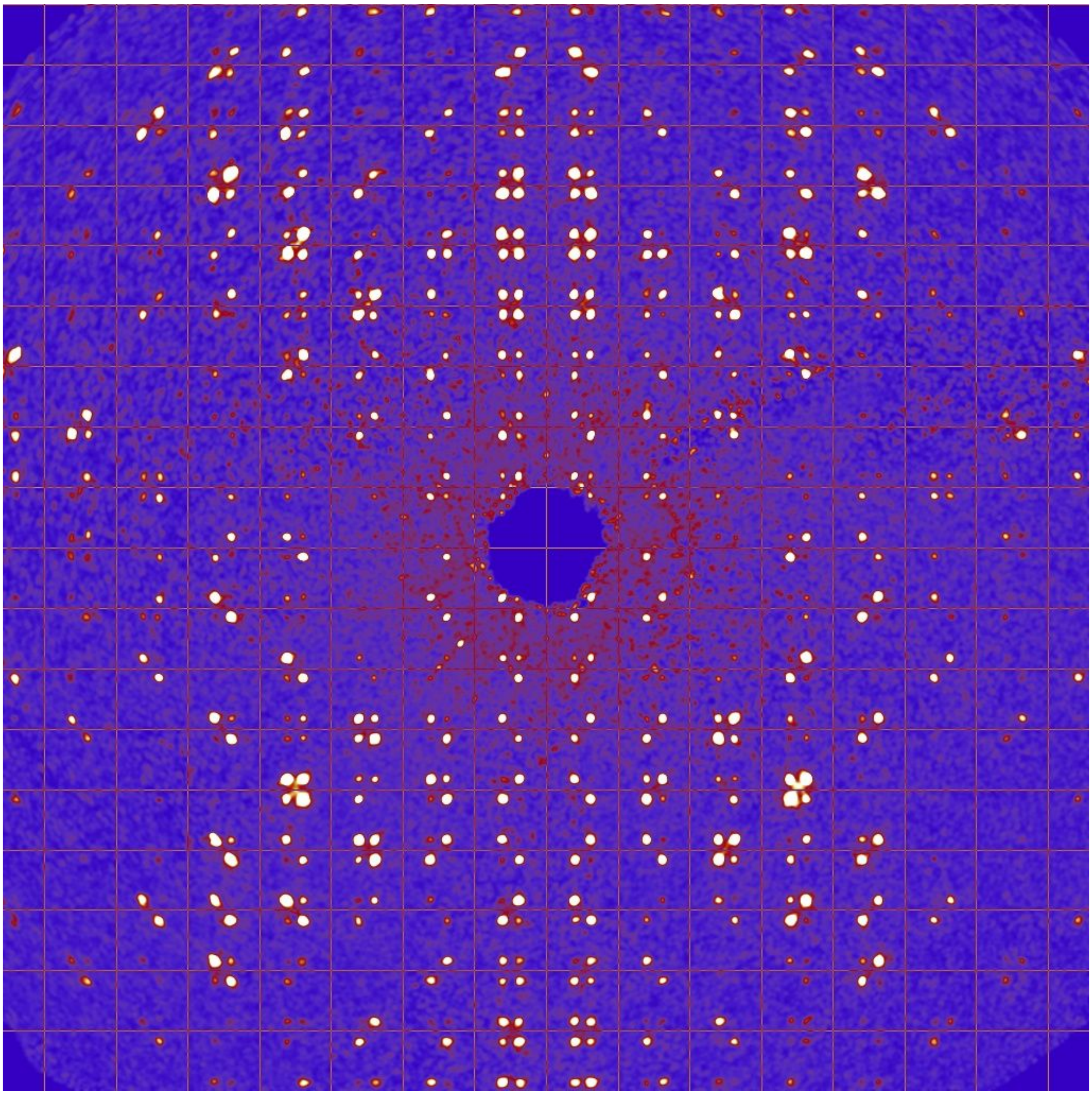

Figure S2. **Section (-1/2 *k l*) of ferrocene phase I'' at 166 K**, calculated from CCD images by CrysAlis^{PRO} instruction *unwrap* of the scXRD diffraction CCD images. The *unwrap* integration range from 0.4*a** to 0.6*a** was applied.

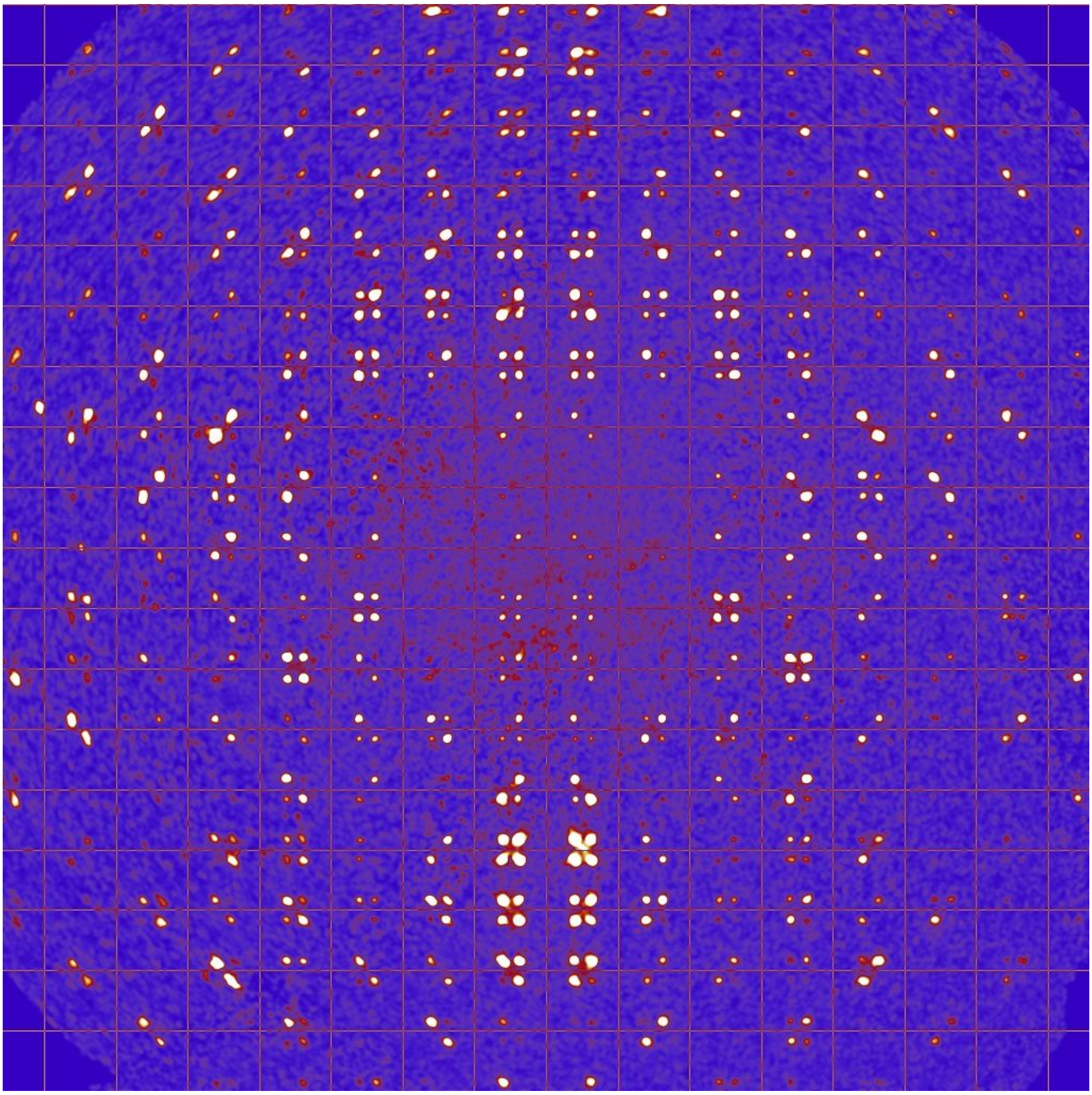

Figure S3. **Section (3/2 *k l*) of ferrocene phase I'' at 166 K**, calculated from CCD images by CrysAlis^{PRO} instruction *unwrap* of the scXRD diffraction CCD images. The *unwrap* integration range from 0.4*a** to 0.6*a** was applied.

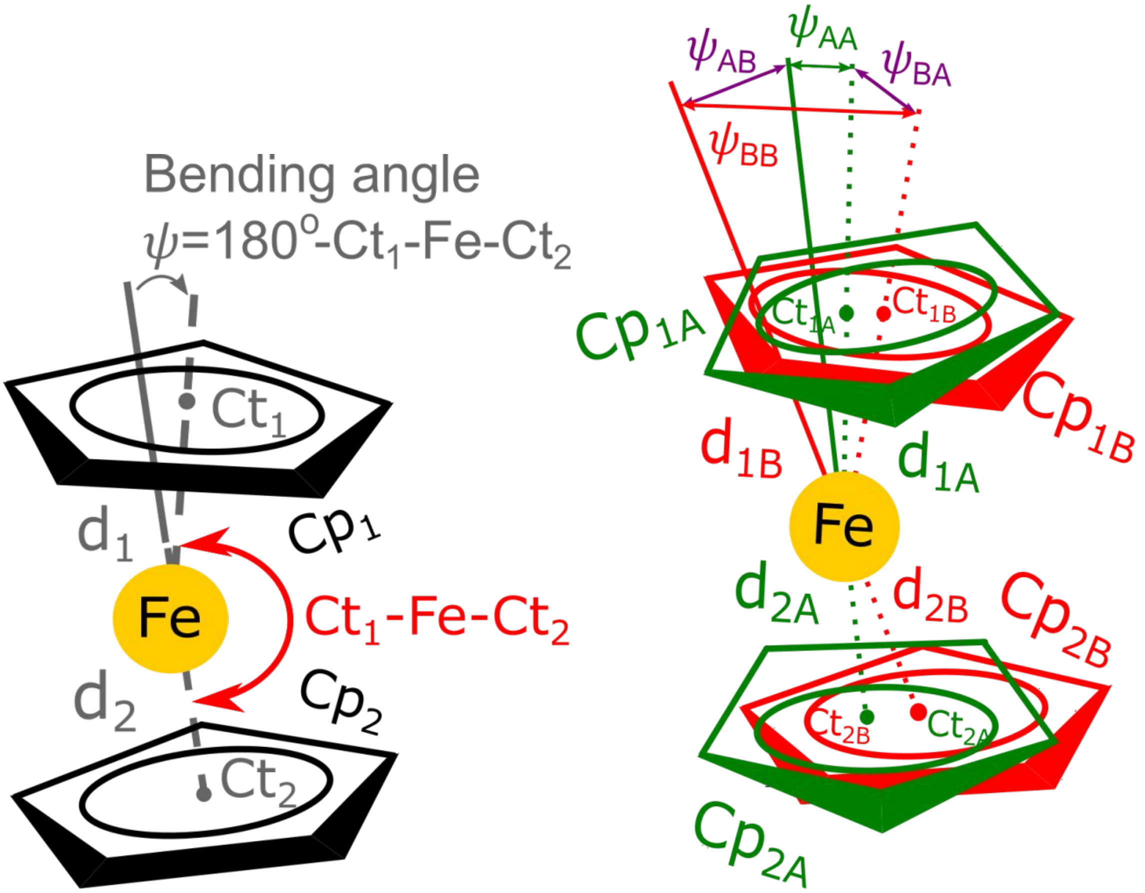

Figure S4. Conformational features of ferrocene molecule in modulated phase I'' relevant to the molecular-dipole generation: (left) bending angle ψ and distances Ct_1—Fe (d_1) and Ct_2—Fe (d_2) for one molecular site; (right) bending angles ψ_{AA}, ψ_{BB}, ψ_{AB} and ψ_{BA} between the disordered Cp rings in sites A (green) and B (red), of directions Ct_{1A}—Fe—Ct_{2A}, Ct_{1B}—Fe—Ct_{2B}, Ct_{1A}—Fe—Ct_{2B} and Ct_{1B}—Fe—Ct_{2A}, respectively. All bending angles, distances d (Ct_{1A}—Fe, Ct_{1B}—Fe, Ct_{2A}—Fe, Ct_{2B}—Fe), Fe^{2+} shifts (Fe_x, Fe_y, Fe_z) from the inversion centre, relative rotations between disordered Cp rings (φ_{1A1B}, φ_{2A2B}) and conformational torsions within and between sites A and B (τ_{1A2A}, τ_{1B2B}, τ_{1A2B}, τ_{1B2A}) are modulated.

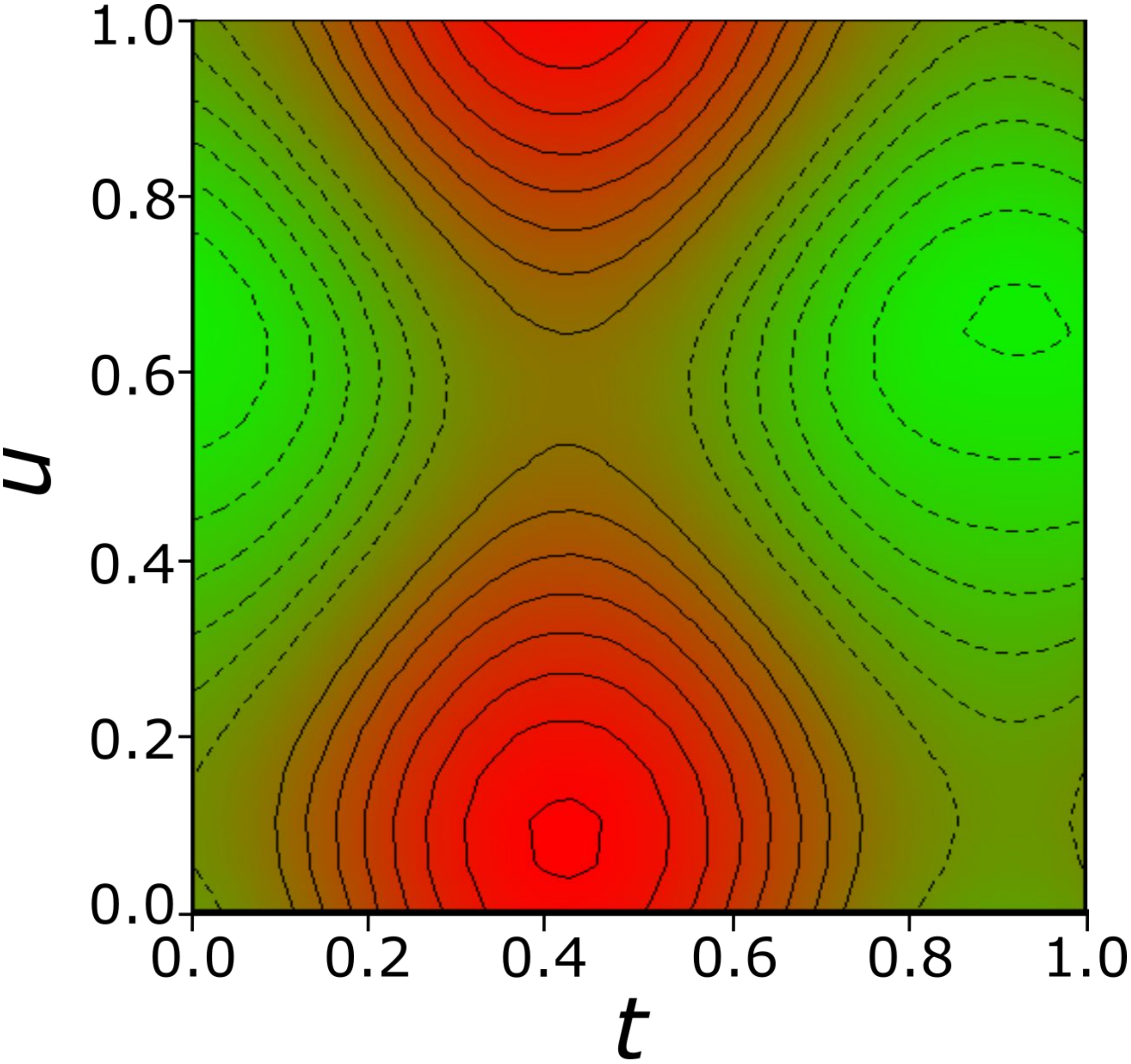

Figure S5. The [t,u]**-modulation map of angle $C1_{1A}$—Fe—$C1_{1B}$** (indices A and B label the sites of the disordered Cp ring, indices 1 and 2 label two Cp rings), measuring the positions of disordered sites. The full and dashed contours mark the excess and deficient values 1° per line, respectively, from the average of 13.65°.

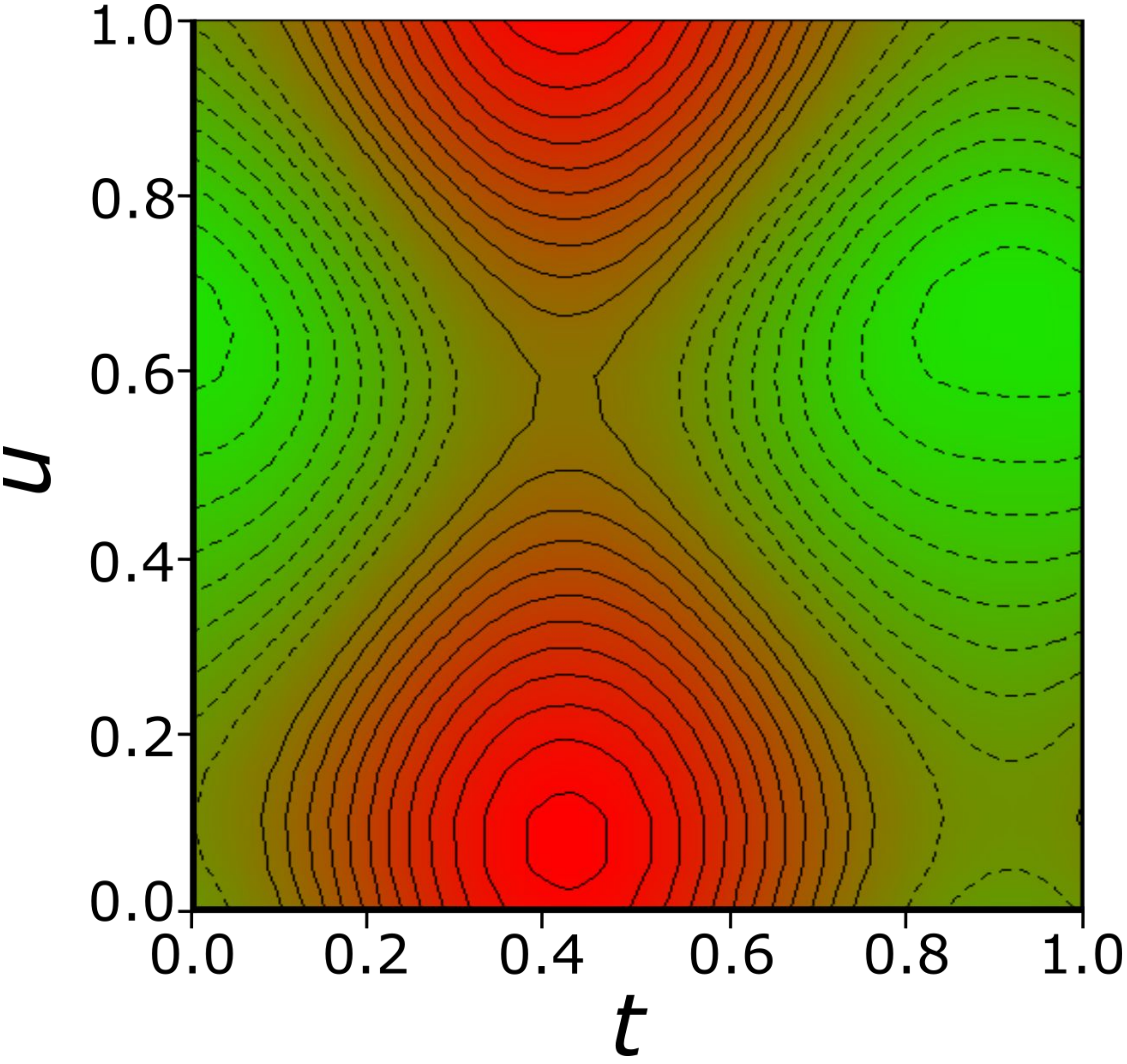

Figure S6. The [*t*,*u*]**-modulation map of distance $C1_{1A}$—$C1_{1B}$** (between sites A and B of the disordered ring Cp_1), illustrating the modulation of half-occupied sites A and B (*cf*. Figure S5). where the full and dashed contours mark the excess and deficient values 0.025 Å per line, respectively, from the average of 0.484 Å.

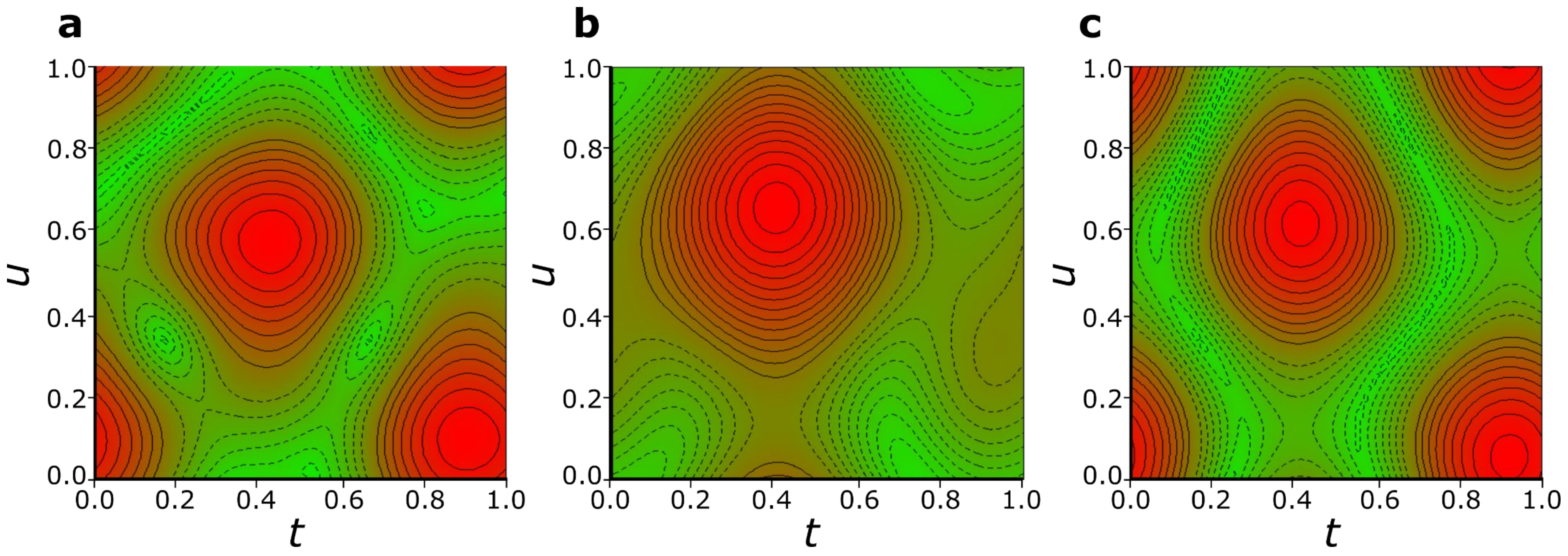

Figure S7. The [t,u]**-modulation map of angles a** $C5_{1A}$—$C1_{1A}\cdots C4_{2A}$; **b** $C5_{1A}$—$C1_{1A}\cdots C4_{2B}$ and **c** $C5_{1B}$—$C1_{1B}\cdots C4_{2B}$ (indices A and B label the sites of the disordered Cp ring, indices 1 and 2 label two Cp rings), where the full and dashed contours mark the excess and deficient values 1° per line, respectively, from the average of **a** 96.5°, **b** 95.3° and **c** 95.9°, the latter between 91.1° and 99.9°.

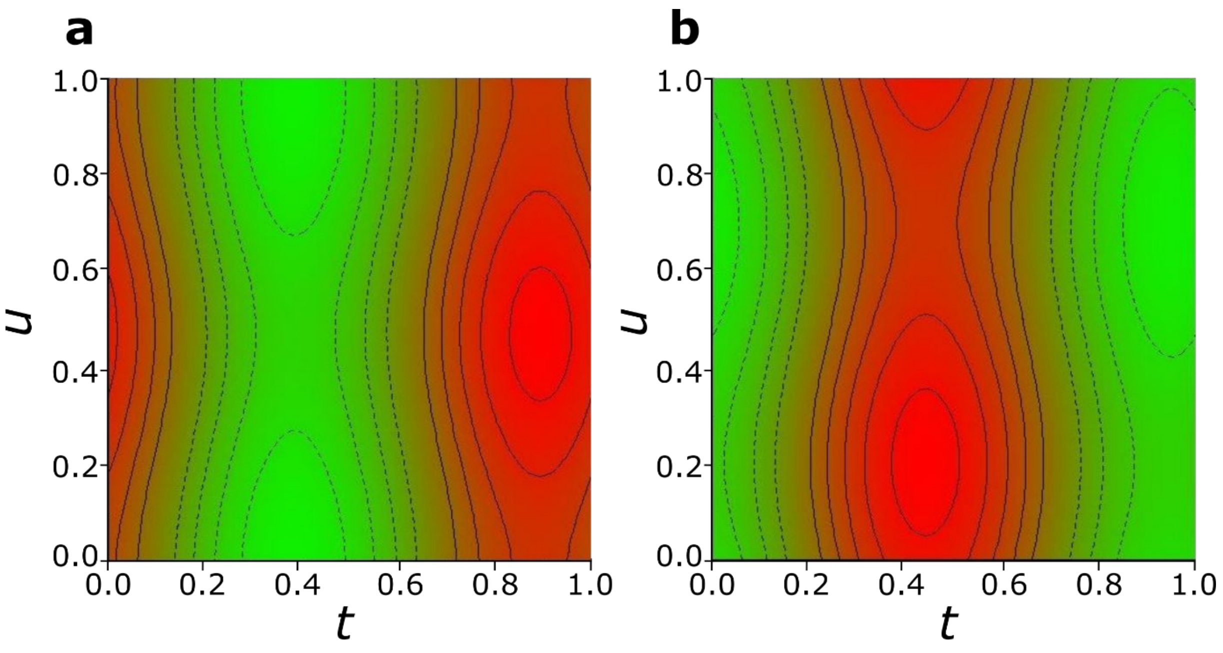

Figure S8. Ring-centroid to Fe dication distances: a Fe—Ct_{1A} and **b** and Fe—Ct_{2A}. The minimum distance is 1.616 Å, average 1.665 Å and maximum 1.720 Å, step 0.01 Å.

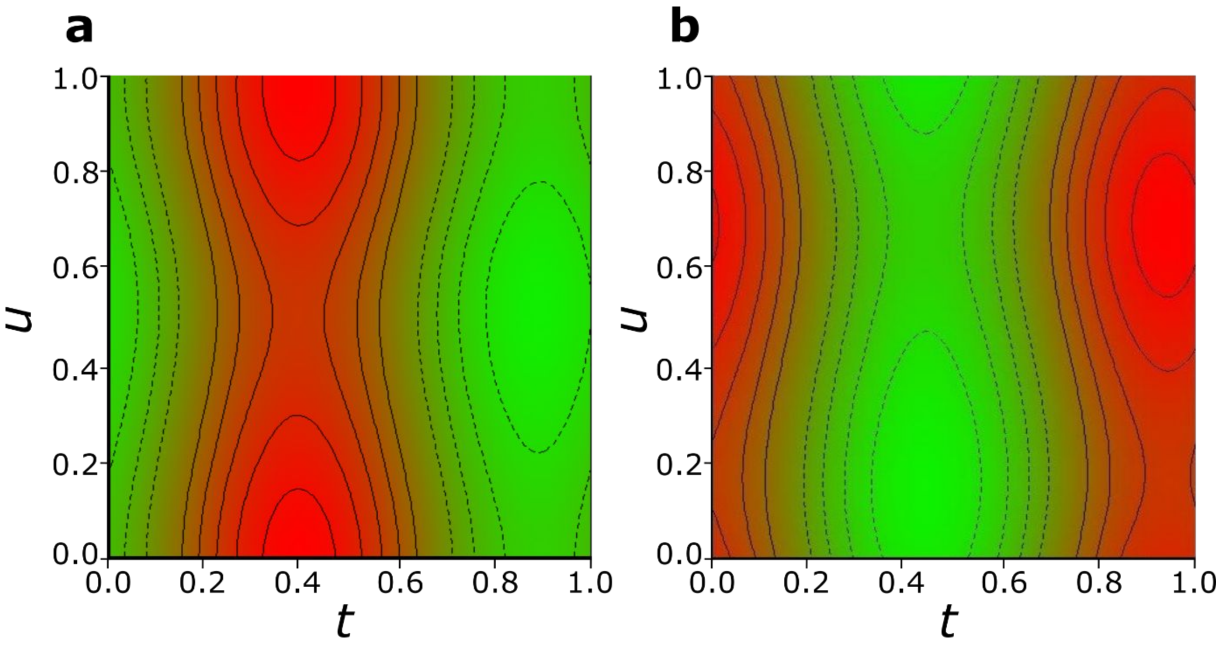

Figure S9. Ring-centroid to Fe dication distances: **a** Fe—Ct_{1B} and **b** Fe—Ct_{2B}. Minimum distance 1.588 Å, average 1.636 Å and maximum 1.692 Å, step 0.01 Å.

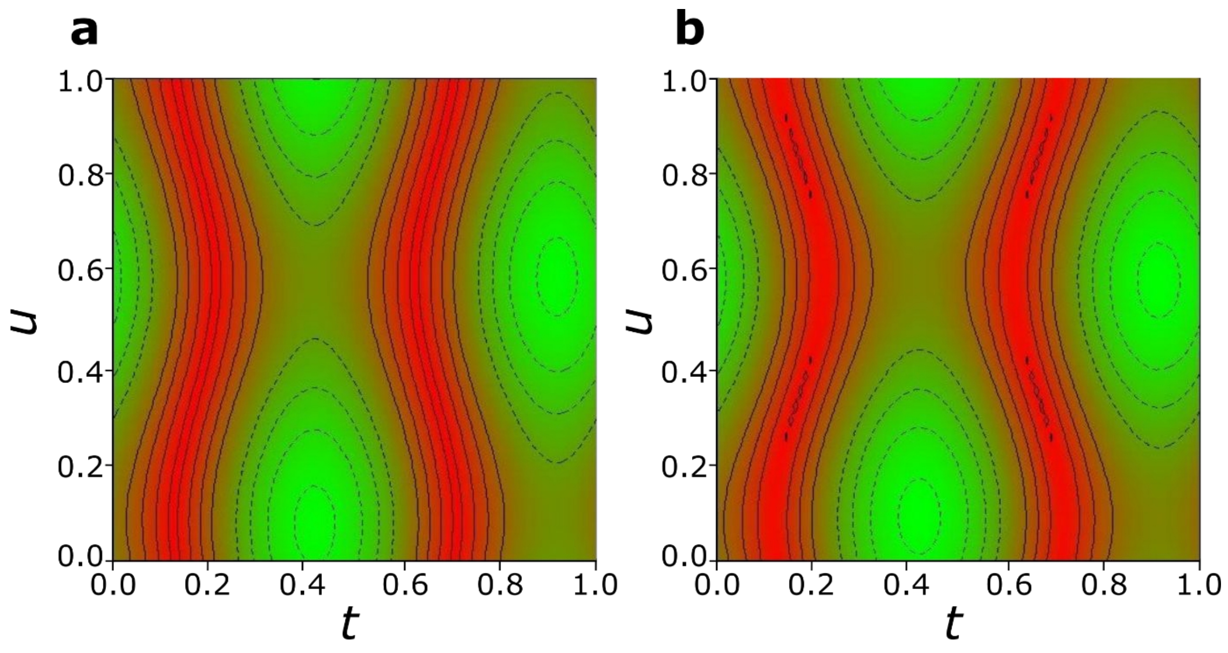

Figure S10. Bending of the molecular axis a Ct_{1A}—Fe—Ct_{2A}: min. 171.38°, aver. 175.60°, max. 179.997°, step 1.00° **b** Ct_{1B}—Fe—Ct_{2B}: min. 171.60°, aver. 175.83°, max. 179.92°, step 1.00°.

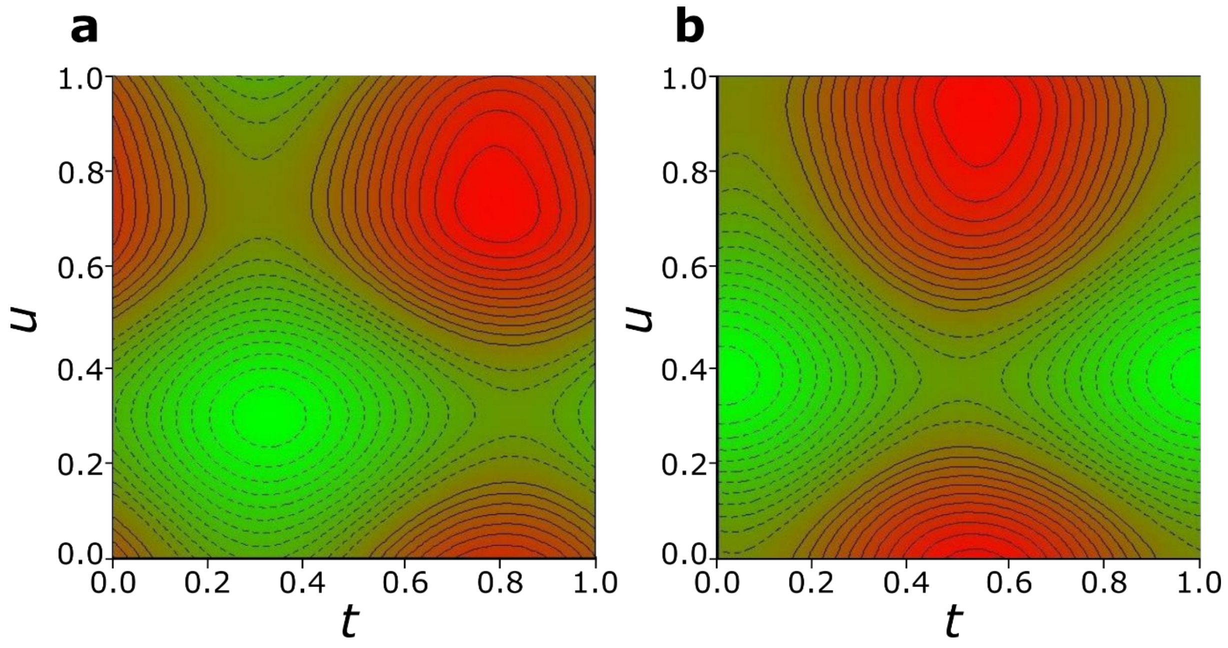

Figure S11. Bending of the molecular axis correlation between sites A and B: **a** angle Ct_{1A}—Fe—Ct_{2B} (min. 173.47°, aver. 176.02°, max. 178.38°, step 0.2°); and **b** angle Ct_{1B}—Fe—Ct_{2A} (min. 173.47°, aver. 176.02°, max. 178.38°, step 0.2°).

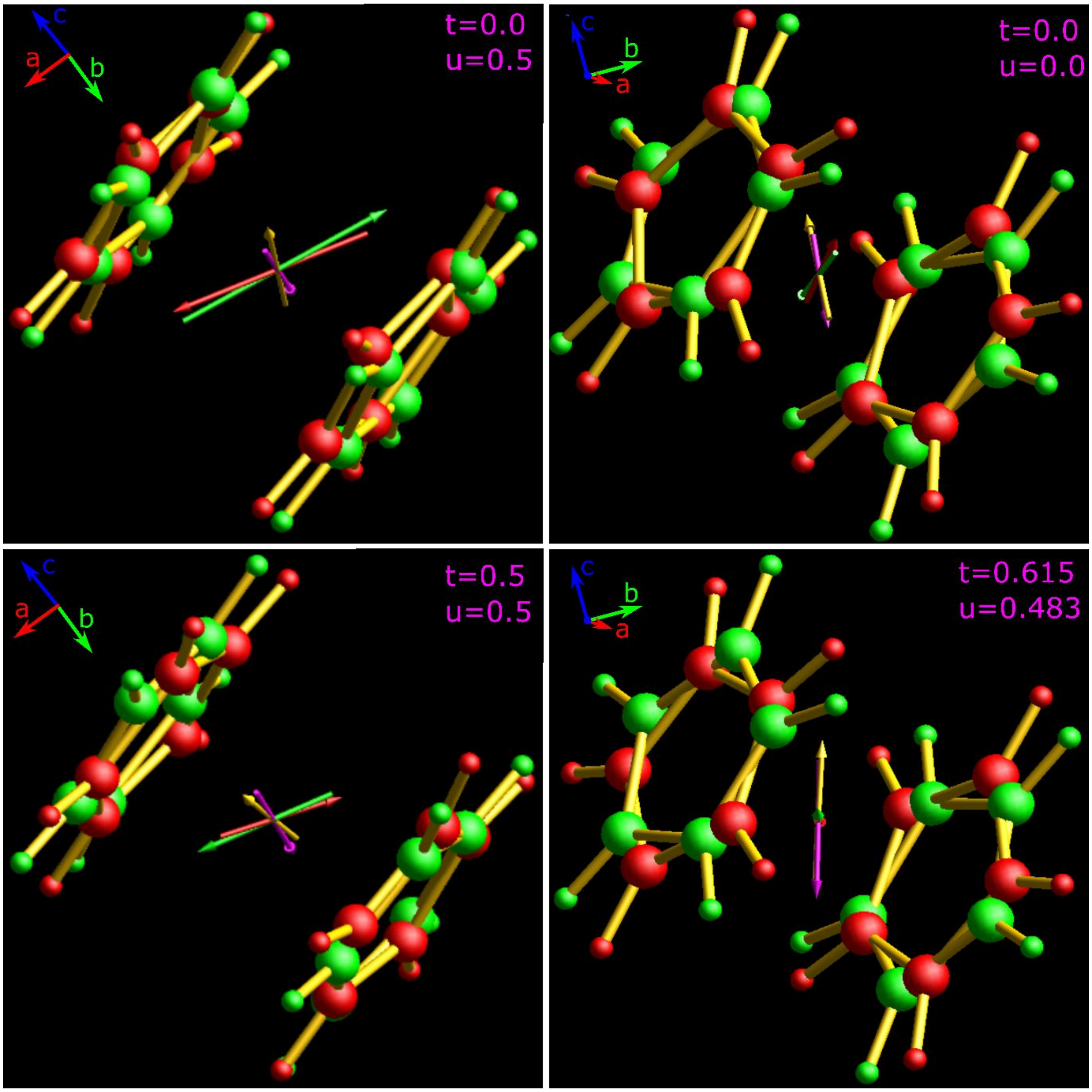

Figure S12. Selected snapshots of the $FeCp_2$ molecule in the incommensurate phase I'' in several different projections, for different [*t*,*u*] modulation coordinates, as specified in the top-left and top-right corners, respectively. Disordered sites A and B are drawn with green and red atoms, respectively. The molecular dipole moments are represented by green, red, purple and yellow arrows for configurations AA, BB, AB and BA, respectively.

Table S1. Crystallographic data of ferrocene phase I'' at 166 K/ 0.1 MPa.

Empirical formula	$C_{10}H_{10}Fe$
M_r	186
Radiation type, λ (Å)	Mo *K*α, 0.71073
Crystal system, space group	Monoclinic, $P2_1/n(\alpha\beta\gamma)00(\alpha\beta\gamma)00^{\dagger}$, 14.2.16.6 ††
Temperature (K)	166
Wave vectors	$q_1 = 0.5000a^* + 0.3880b^* + 0.1660c^*$;<br>$q_2 = -0.5000a^* + 0.3880b^* -0.1660c^*$
a, *b*, *c* (Å)	5.7982 (2), 7.5645 (3), 8.9915 (4)
β (°)	92.911 (3)
V (Å³)	393.86 (3)
Z	2
Calculated density (g/cm³)	1.569
Absorption coefficient (mm⁻¹)	1.834
F(000)	192
Crystal size (mm)	0.55/0.41/0.32
θ-range for data collection (°)	4.09/28.22
Diffractometer	Xcalibur, Atlas, Gemini ultra
Absorption correction	Empirical absorption correction using spherical harmonics<br>implemented in CrysAlis PRO †††
T_{min}, T_{max}	0.83, 1
No. of measured, independent and<br>observed [$I > 3\sigma(I)$] reflections	38901, 4755, 2360
R_{int}	0.028
$(\sin \theta/\lambda)_{max}$ (Å⁻¹)	0.672
Refinement method	Full-matrix least-squares on F^2's
$R_{all}[F^2 > 3\sigma(F^2)]$, $wR(F^2)$, S	0.035, 0.091, 1.14
$R_{main}[F^2 > 3\sigma(F^2)]$, $wR(F^2)$	0.025, 0.067
$R_{sat}[F^2 > 3\sigma(F^2)]$, $wR(F^2)$	0.069, 0.019
No. of reflections	4755
No. of parameters	149
H-atom treatment	H-atom parameters constrained
$\Delta\rho_{max}$, $\Delta\rho_{min}$ (e Å⁻³)	0.52, −0.67

† Symmetry operations: (1) x1, x2, x3, x4, x5; (2) −x1+1/2, x2+1/2, −x3+1/2, x5+1/2, x4+1/2; (3) −x1, −x2, −x3, −x4, −x5; (4) x1+1/2, −x2+1/2, x3+1/2, −x5+1/2, −x4+1/2.

†† https://stokes.byu.edu/iso/findssg.php

††† CrysAlis PRO 1.171.40.53 (Rigaku OD, 2019).

Table S2. Structural parameters of ferrocene phase I'' at 166 K/0.1 MPa.

Positional parameters of individual atoms							
	occ		x	y	z	U_{iso}	
Fe1	1		0.5	0	0.5	0.02352(8)	
		s,1,0 †	-0.00018(4)	0.00295(3)	0.00842(3)		
		c,1,0	0	0	0		
		s,0,1	-0.00245(4)	0.00432(3)	-0.00214(3)		
		c,0,1†	0	0	0		
Positional parameters of atoms described as a rigid body							
C1	1		0.4661	0.2629	0.4731		
C2	1		0.2415(4)	0.2014(5)	0.4806(4)		
C4	1		0.4050(9)	0.0592(6)	0.2921(4)		
C3	1		0.2029(8)	0.0752(6)	0.3689(5)		
C5	1		0.5670(6)	0.1752(5)	0.3570(3)		
H1c1	1		0.538(3)	0.3480(18)	0.5346(17)		
H1c2	1		0.138(3)	0.234(2)	0.5552(15)		
H1c4	1		0.429(3)	-0.0168(19)	0.2143(18)		
H1c3	1		0.062(3)	0.006(2)	0.351(2)		
H1c5	1		0.711(2)	0.191(2)	0.3307(17)		
ADP harmonic parameters of individual atoms							
		U_{11}	U_{22}	U_{33}	U_{12}	U_{13}	U_{23}
Fe1		0.02633(14)	0.01874(13)	0.02531(13)	0.00138(7)	-0.00049(9)	0.00417(7)
	s,1,0	0	0	0	0	0	0
	c,1,0	0.00000(15)	-0.00030(14)	-0.00090(13)	0.00072(12)	0.00109(11)	-0.00007(11)
	s,0,1	0	0	0	0	0	0
	c,0,1	-0.00016(15)	0.00030(15)	0.00175(13)	0.00012(12)	0.00060(11)	0.00064(11)
Positional parameters of the rigid body ††							
		φ	χ	ψ	x-trans	y-trans	z-trans
Position 1		0	0	0	0.0323(6)	0.0018(4)	-0.0135(4)
	s,1,0	0.0121(4)	-0.0190(3)	0.0159(2)	-0.0502(6)	0.0053(3)	0.0177(3)
	c,1,0	-0.0089(4)	0.0140(2)	-0.0128(2)	0.0378(5)	-0.0031(3)	-0.0131(3)
	s,0,1	0.0059(4)	-0.0083(3)	0.0070(3)	-0.0230(6)	0.0045(4)	0.0048(3)
	c,0,1	-0.0014(4)	0.0087(3)	-0.0037(3)	0.0125(6)	-0.0044(4)	-0.0027(3)
Position 2		11.5(2)	-13.4(2)	6.0(3)	-0.0302(5)	-0.0003(4)	0.0155(3)
	s,1,0	0.0084(4)	-0.0206(2)	0.0128(2)	-0.0157(4)	0.0070(3)	0.0215(3)
	c,1,0	-0.0044(4)	0.0079(2)	-0.0059(2)	0.0097(4)	-0.0041(3)	-0.0104(3)
	s,0,1	0.0043(4)	-0.0017(3)	0.0011(2)	-0.0002(4)	0.0045(4)	0.0049(3)
	c,0,1	-0.0002(4)	0.0012(3)	-0.0018(2)	0.0044(4)	-0.0016(4)	0.0038(3)
TLS parameters of the rigid body †††							
Position 1	T	0.0265(9)	0.0071(5)	0.0055(3)	-0.0004(5)	0.0001(5)	0.0016(2)
	L	0.0037(17)	0.0035(6)	0.0032(5)	0.0017(5)	-0.0004(3)	-0.0004(4)
	S	0.0020(16)	-0.0017(10)	0.0013(8)	0.0033(5)	-0.0006(6)	0.0006(3)
		-0.0061(5)	0.0014(4)	-0.0006			
Position 2	T	0.0157(6)	0.0075(4)	0.0073(4)	-0.0036(4)	-0.0013(4)	0.0026(3)
	L	0.0011(13)	0.0028(5)	0.0023(4)	-0.0004(4)	0.0006(3)	-0.0019(4)
	S	-0.0001(13)	0.0013(8)	0.0018(7)	0.0004(4)	-0.0016(5)	-0.0005(3)
		-0.0037(4)	0.0016(4)	0.0011			

† „s,1,0“ means sinus wave with the wave vector $1\mathbf{q_1}+0\mathbf{q_2}$; „c,0,1“ means cosinus wave with the wave vector $0\mathbf{q_1}+1\mathbf{q_2}$

†† V. Petricek, P. Coppens, P. Becker (1985). Structure Analysis of Displacively Modulated Molecular Crystals. *Acta Cryst*. A41, 478-483.

††† V. Schomaker, K.N. Trueblood (1968) *Acta Cryst*., B24, 63 - 76.

Supplementary Videos Titles:

Video S1. Animation of the ferrocene molecule in phase I'' viewed perpendicular to pseudo-D_5 axis. The animation shows positions and displacement parameters of ferrocene as a function of the modulation coordinates [*t*, *u*], as indicated in the top-right corner. Both disordered sites A and B with equal site-occupation factors (SOF=0.5) are shown; the colour code of atoms: Fe gold, C brown and H white. The Fe and C atoms are represented at the 30% probability level; H atoms are shown as small white spheres.

Video S2. Animation of the ferrocene molecule in phase I'' viewed along the pseudo-D_5 axis. The animation shows positions and displacement parameters of ferrocene as a function of the modulation coordinates [*t*, *u*], as indicated in the top-right corner. Both disordered sites A and B with equal site-occupation factors (SOF=0.5) are shown; the colour code of atoms: Fe gold, C brown and H white. The Fe and C atoms are represented at the 30% probability level; H atoms are shown as small white spheres.

Video S3. Animation of the dipole moments of disordered sites of ferrocene molecule in phase I'' in a side view. The animation shows positions, displacement parameters and dipole moment of ferrocene as a function of the modulation coordinates [*t*, *u*], as indicated in the top-right corner. The Cp rings disordered in sites A are shown as green spheres and in sites B are coloured red; the dipole moment of configuration $Cp_{1A}FeCp_{2A}$ is marked green, that of configuration $Cp_{1B}FeCp_{2B}$ is red, that of $Cp_{1A}FeCp_{2B}$ purple and that of configuration $Cp_{1B}FeCp_{2A}$ is yellow. The amplitudes of the dipole moment modulations for these configurations are 1.30 D, 1.27 D, 1.04 D and 1.04 D, respectively. The dipole-moment vectors $\boldsymbol{\mu}$ can be decomposed in the molecular coordinates into components [μ_h, μ_v]: horizontal component μ_h (perpendicular to the pseudo-D_5 axis, derived from the angle Ct_1–Fe–Ct_2 bending); and vertical component μ_v (along the pseudo-D_5 axis, derived from the difference between Ct_1–Fe and Fe–Ct_2 distances). These amplitude components for the dipole moments are: $\boldsymbol{\mu}_{1A2A}$=[1.19, 0.53] D; $\boldsymbol{\mu}_{1B2B}$=[1.16, 0.51] D; $\boldsymbol{\mu}_{1A2B}$=[0.90, 0.52] D and $\boldsymbol{\mu}_{1B2A}$=[0.90, 0.52] D.

Video S4. Animation of the dipole moments of disordered sites of ferrocene molecule in phase I'' viewed along the pseudo-D_5 axis – *cf.* the caption of Video S3.

Video S5. Animation of the dipole moments of disordered sites of ferrocene molecule in phase I'' viewed perpendicular to the molecular pseudo-D_5 axis – *cf.* the caption of Video S3.
