# Supplementary material for: Dipole-Moment Modulation in New Incommensurate Ferrocene
Source: J Phys Chem Lett. 2023 Mar 23;14(13):3111–9. doi: 10.1021/acs.jpclett.3c00215 (PMC10084461; doi:10.1021/acs.jpclett.3c00215)
Supplement: Supplementary file 7 — jz3c00215_si_007.pdf [file jz3c00215_si_007.pdf]

jz-2023-00215f.R1

Name: Peer Review Information for "Dipole-Moment Modulation in New Incommensurate Ferrocene"

First Round of Reviewer Comments

Reviewer: 1

Comments to the Author

I enjoyed reading this manuscript. The work seems to have been done, reported, and referenced very carefully.

Ferrocene is one of the small number of simple, classic molecules that have been studied intensively for decades. It has been clear for a long time that something important about the solid phases of ferrocene was not yet understood. This work is therefore a very important contribution.

The fact that the structure of the new phase cannot be described without a second incommensurate modulation vector is very interesting. I don't think many such structures have been refined and reported. Perhaps more could be made of that fact. How many such structures are known?

Re Fig 3: How were pairings of the two rings (ie, the "possible configurations") determined so as to give the distorted molecular structures shown at the bottom of the figure? I think this question needs to be answered briefly in the text and in more detail in the supplementary material.

The description of the modulated phase will be heavy going even for experienced crystallographers. Could it be simplified with the full version moved to the supplementary material?

Minor comments:

Pg 1, line 53L Should 32 deg be changed to 36 deg?

Pg 1, line 56L The reference should be changed from "18, 19" to "19" because reference 18 includes no crystallographic data.

Fig. 1 I found this figure confusing. In the two lower images the colors have been switched relative to the colors in the upper image. That inconsistency needs to be fixed.

Pg 2, line 8R Recommend changing "molecules display some departures from" to "molecules necessarily display some departures from" because departures as small as are describe could easily arise from the absence of fivefold symmetry in the molecule's surroundings.

Pg 2, line 18R Recommend changing "are not parallel" to "are not quite parallel" because the angular deviations are so small.

Pg 2, line 33R Recommend changing "so far unknown" to "previously unknown".

Pg 2, line 45R Recommend changing “then neglected” to “previously neglected”.

Pg 3, line 4L The word “exemplary” is a problem because in at least some parts of the of the world it usually appears in a different context (eg, His conduct was exemplary.). The word “Sample” might be a suitable replacement.

Pg 3, line 18Lff What are the estimated uncertainties of these thermodynamic values? How many of the digits are significant?

Fig 2 caption Please change “consequtive” to “consecutive”.

Pg 4, lines 46-48R The subject “amplitudes” and verb “is” do not agree.

Pg 5, lines 8-9R The subject “feature” and verb “are” do not agree..

Pg 5, line 11Rff Again, I think estimated standard deviations should be given.

Pg 5, line 47R Should the “-“ in “Cp-“ be made a superscript?

Ref 1 “Compoun” needs to be changed to “Compound”.

Ref 60 A paragraph mark needs to be inserted before ‘(60)”.

Fig S1-S3 The word “CrystAlice” is almost certainly wrong. Should it be “CrysAlis”?

Reviewer: 2

Comments to the Author

Comments for Authors on Manuscript jz-2023-00215f

This manuscript by Katrusiak *et al.* reports exciting new results, uncovering a previously undetected modulated phase of ferrocene, and clarifies a number of puzzling anomalies, focusing on those involving dielectric properties. This advance constitutes an important extension of our understanding of the ferrocene phase diagram and may well lead to additional insights for related metallocenes in the solid state.

The quantitative description of ferrocene thermal properties and dielectric response achieved here furthers our understanding of adsorption properties and interactions with solvents for this archetypical organometallic compound. The conclusions are fully supported by the data. I strongly recommend publication in *J. Phys. Chem. Lett.*

I have a number of technical suggestions, as follows:

1. The historical introduction is excellent and provides a good overview for non-specialist readers. It may be desirable to add a citation to the pioneering work of E.O. Fischer and W. Pfab (1952).

2. Again, in the Introduction, first paragraph, last sentence, the next-to-last line should read, "...pseudo D_5 <u>axes</u>..." (plural axes).

3. On page 2, first paragraph, second column, line 11 should read, "…ordered phase I'… (replace I'' by I').

4. On page 5, second column, first paragraph, the second sentence should read, “Another molecular feature of ferrocene phase I'' is significantly non-parallel Cp rings.”

5. In the caption for Figure 2, second line, consequtive [sic] is misspelled. On line 5 of the caption, the text should read, “for coarse powder B and…”.

6. If possible, the layout should be adjusted such that captions are placed directly below the figures in question and are contiguous, i.e., do not flow onto the adjoining column of text (Figures 3 and 6 are affected).

Author's Response to Peer Review Comments:

Dear Prof. Editor,

Thank you for your letter and for the Reveiwers' reports on our submission jz-2023-00215f:

Title: "Dipole-Moment Modulation in New Incommensurate Ferrocene"
Authors: Katrusiak, Andrzej; Rusek, Michalina; Dusek, Michal; Petricek, Vaclav; Szafrański, Marek

We have revised the manuscript following the comments from the Referees.

We are grateful to Referees 1 and 2 for carefully reading our manuscript and for their comments. In the PDF accompanying the revision manuscript the revisions are highlighted yellow. Below are our responses to Referees' queries.

We have also prepared a suggestion of the cover graphics based on our article, which is attached to this resubmission. Unfortunately, we cannot consider the complementary cover due to our financial constraints.

With kind regards,

Andrzej Katrusiak

Responses to Referees' comments:

Referee 1

I enjoyed reading this manuscript. The work seems to have been done, reported, and referenced very carefully.

Ferrocene is one of the small number of simple, classic molecules that have been studied intensively for decades. It has been clear for a long time that something important about the solid phases of ferrocene was not yet understood. This work is therefore a very important contribution.

The fact that the structure of the new phase cannot be described without a second incommensurate modulation vector is very interesting. I don't think many such structures have been refined and reported. Perhaps more could be made of that fact. How many such structures are known?

Authors respond: We are grateful for indicating this subject – we have checked

Re Fig 3: How were pairings of the two rings (ie, the "possible configurations") determined so as to give the distorted molecular structures shown at the bottom of the figure? I think this question needs to be answered briefly in the text and in more detail in the supplementary material.

The description of the modulated phase will be heavy going even for experienced crystallographers. Could it be simplified with the full version moved to the supplementary material?

Authors respond: We are grateful for this comment – we have better, we believe, described the modulation and the ferrocene structure, and particularly focused this description on the inclination of the Centroid1-Fe-Centroid2 directions. This is the most important parameter of the molecule responsible for its dipole moment and the bending of this angle is the main new conformational property revealed in our paper.

Minor comments:

Pg 1, line 53L Should 32 deg be changed to 36 deg?

Authors respond: We are grateful for indicating this error – we have corrected it now.

Pg 1, line 56L The reference should be changed from "18, 19" to "19" because reference 18 includes no crystallographic data.
Authors respond: We are grateful for indicating this mistake – indeed reference 18 proposed the sandwich structure, which was quite independently confirmed by 3 groups by X-ray diffraction. We have now corrected this inconsistence and added the appropriate 3 papers, while we have better described the achievement of reference 18.
Fig. 1 I found this figure confusing. In the two lower images the colors have been switched relative to the colors in the upper image. That inconsistency needs to be fixed.
Authors respond: We are grateful for indicating this mistake – we have corrected it in the revised version.
Pg 2, line 8R Recommend changing "molecules display some departures from" to "molecules necessarily display some departures from" because departures as small as are describe could easily arise from the absence of fivefold symmetry in the molecule's surroundings.
Authors respond: We are grateful for this comment – indeed it makes the understanding of our intention easier.
Pg 2, line 18R Recommend changing "are not parallel" to "are not quite parallel" because the angular deviations are so small.
Authors respond: We are grateful for this comment – it improves the understanding.
Pg 2, line 33R Recommend changing "so far unknown" to "previously unknown".
Authors respond: We are grateful for this indication – we have changed it.
Pg 2, line 45R Recommend changing "then neglected" to "previously neglected".
Authors respond: We are grateful for this indication – we have changed it.
Pg 3, line 4L The word "exemplary" is a problem because in at least some parts of the of the world it usually appears in a different context (eg, His conduct was exemplary.). The word "Sample" might be a suitable replacement.
Authors respond: We are grateful for this indication – we have applied it.
Pg 3, line 18Lff What are the estimated uncertainties of these thermodynamic values? How many of the digits are significant?
Authors respond: We are grateful for this comment – we have calculated precisely the estimated standard deviations and included them throughout the text.
Fig 2 caption Please change "consequtive" to "consecutive".
Authors respond: We are grateful for indicating this mistake – we have corrected it.
Pg 4, lines 46-48R The subject "amplitudes" and verb "is" do not agree.
Authors respond: We are grateful for this indication – we have corrected it.
Pg 5, lines 8-9R The subject "feature" and verb "are" do not agree..
Authors respond: We are grateful for this indication – we have corrected it.
Pg 5, line 11Rff Again, I think estimated standard deviations should be given.
Authors respond: We are grateful for this indication – we have added the standard deviations to all experimentally measured values. In the case of calorimetric values it is generally accepted that the estimated standard deviations are at the order of the last significant digids.
Pg 5, line 47R Should the "-" in "Cp-" be made a superscript?
Authors respond: We are grateful for this indication – we have corrected it.
Ref 1 "Compoun" needs to be changed to "Compound".
Authors respond: We are grateful for this indication – we have corrected it.

Ref 60 A paragraph mark needs to be inserted before '(60)".
Authors respond: We are grateful for this indication – we have corrected it.
Fig S1-S3 The word "CrystAlice" is almost certainly wrong. Should it be "CrysAlis"?
Authors respond: We are grateful for this indication – we have corrected it.

Comments for Authors on Manuscript jz-2023-00215f
This manuscript by Katrusiak et al. reports exciting new results, uncovering a previously undetected modulated phase of ferrocene, and clarifies a number of puzzling anomalies, focusing on those involving dielectric properties. This advance constitutes an important extension of our understanding of the ferrocene phase diagram and may well lead to additional insights for related metallocenes in the solid state.
The quantitative description of ferrocene thermal properties and dielectric response achieved here furthers our understanding of adsorption properties and interactions with solvents for this archetypical organometallic compound. The conclusions are fully supported by the data. I strongly recommend publication in J. Phys. Chem. Lett.
Authors respond: We are very grateful for this favourable assessment of our work.

I have a number of technical suggestions, as follows:
1. The historical introduction is excellent and provides a good overview for non-specialist readers. It may be desirable to add a citation to the pioneering work of E.O. Fischer and W. Pfab (1952).
Authors respond: We are grateful for indicating this missing reference, which has been added in the revised manuscript.
2. Again, in the Introduction, first paragraph, last sentence, the next-to-last line should read, "...pseudo D5 axes..." (plural axes).
Authors respond: We are grateful for indicating this mistake, it has been corrected.
3. On page 2, first paragraph, second column, line 11 should read, "...ordered phase I'... (replace I" by I').
Authors respond: We are grateful for indicating this mistake, now corrected.

4. On page 5, second column, first paragraph, the second sentence should read, "Another molecular feature of ferrocene phase I" is significantly non-parallel Cp rings."
Authors respond: We are grateful for indicating this mistake, now corrected.

5. In the caption for Figure 2, second line, consequtive [sic] is misspelled. On line 5 of the caption, the text should read, "for coarse powder B and...".
Authors respond: We are grateful for indicating these mistakes, now corrected.

6. If possible, the layout should be adjusted such that captions are placed directly below the figures in question and are contiguous, i.e., do not flow onto the adjoining column of text (Figures 3 and 6 are affected).
Authors respond: We are grateful for indicating this problem – we have tried to cure it.
